# Anthropogenic fragmentation increases risk of genetic decline in the threatened orchid *Platanthera leucophaea*

**DOI:** 10.1002/ece3.8578

**Published:** 2022-02-17

**Authors:** Claire Ellwanger, Laura Steger, Cathy Pollack, Rachel Wells, Jeremie Benjamin Fant

**Affiliations:** ^1^ 5170 Plant Biology and Conservation Chicago Botanic Garden Glencoe Illinois USA; ^2^ Plant Biology and Conservation Northwestern University, O.T. Hogan Hall Evanston Illinois USA; ^3^ U.S. Forest Service Okanogan‐Wenatchee National Forest Wenatchee Washington USA; ^4^ 7864 School of Life Sciences Arizona State University Tempe Arizona USA; ^5^ U.S. Fish and Wildlife Service Chicago Field Office Chicago Illinois USA; ^6^ 5170 Department of Biology University of Louisville Louisville Kentucky USA

**Keywords:** conservation, fragmentation, gene flow, inbreeding, orchids, population genetics, rare species

## Abstract

Protecting biodiversity requires an understanding of how anthropogenic changes impact the genetic processes associated with extinction risk. Studies of the genetic changes due to anthropogenic fragmentation have revealed conflicting results. This is likely due to the difficulty in isolating habitat loss and fragmentation, which can have opposing impacts on genetic parameters. The well‐studied orchid, *Platanthera leucophaea*, provides a rich dataset to address this issue, allowing us to examine range‐wide genetic changes. Midwestern and Northeastern United States. We sampled 35 populations of *P. leucophaea* that spanned the species’ range and varied in patch composition, degree of patch isolation, and population size. From these populations we measured genetic parameters associated with increased extinction risk. Using this combined dataset, we modeled landscape variables and population metrics against genetic parameters to determine the best predictors of increased extinction risk. All genetic parameters were strongly associated with population size, while development and patch isolation showed an association with genetic diversity and genetic structure. Genetic diversity was lowest in populations with small census sizes, greater urbanization pressures (habitat loss), and small patch area. All populations showed moderate levels of inbreeding, regardless of size. Contrary to expectation, we found that critically small populations had negative inbreeding values, indicating non‐random mating not typically observed in wild populations, which we attribute to selection for less inbred individuals. The once widespread orchid, *Platanthera leucophaea*, has suffered drastic declines and extant populations show changes in the genetic parameters associated with increased extinction risk, especially smaller populations. Due to the important correlation with risk and habitat loss, we advocate continued monitoring of population sizes by resource managers, while the critically small populations may need additional management to reverse genetic declines.

## Graphical abstract

Protecting biodiversity requires an understanding of how anthropogenic changes impact the genetic processes associated with extinction risk. The well‐studied orchid, *Platanthera leucophaea*, provides a rich dataset to evaluate the potentially opposing impact of habitat loss and fragmentation on genetic parameters, allowing us to examine range‐wide genetic changes. The once widespread orchid has suffered drastic declines and extant populations show changes in the genetic parameters associated with increased extinction risk, especially in smaller populations.

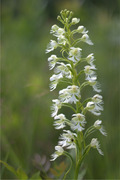

## 1 INTRODUCTION

Anthropogenic‐driven fragmentation is a major threat to biodiversity worldwide (Lienert, 2004). Over time, structural changes to habitat reduces patch size while increasing proximity to human‐modified landscapes and isolation between populations (Haddad et al., 2015). These interwoven landscape effects can operate over potentially long timescales to drive declines in both species and genetic diversity (Haddad et al., 2015; Ibáñez et al., 2014). Identifying the factors that most impact species decline can help managers prioritize conservation practices. There is debate over the relative importance of different types of habitat changes in driving biodiversity losses (Fahrig, 2017; Fahrig et al., 2019; Fletcher et al., 2018; Hadley & Betts, 2016). Although loss of habitat alone can explain biodiversity loss (Fahrig, 2017), patterns of biodiversity change are often explained by the complex interactions between patch area declines, connectivity reductions, and increased edge effects (Fletcher et al., 2018; Haddad et al., 2015). Given that genetic factors are one of the major causes of species extinction (Frankham, 2005), evaluating the impacts of anthropogenic changes on species success is critical to conservation efforts (Leimu et al., 2010).

The local extinction of a plant species is typically driven my multiple interacting factors. Under anthropogenic changes to landscapes such factors can include changes in habitat suitability (Breed et al., 2012), increased competition from invasion (edge effects; González‐Varo et al., 2012), loss of symbionts (mycorrhizae, pollinators; Broadhurst & Young, 2006; Jacquemyn et al., 2004), reduced recruitment (González‐Varo et al., 2012), loss of habitat area, and increased patch isolation (Butaye et al., 2001; Honnay et al., 2002). Together these changes negatively impact the demographic trajectory and genetic diversity, and ultimately reduce the reproductive output of plant populations (Aguilar et al., 2006, 2008; Angeloni et al., 2011; Honnay & Jacquemyn, 2007b; Leimu et al., 2006; Vranckx et al., 2011). These factors work together to accelerate decline, spiraling a population in a downward trajectory known as the “extinction vortex” (Gilpin & Soule, 1989). This increased risk is in part driven by genetic changes to the population during habitat fragmentation including increased inbreeding, loss of genetic variability, and increased divergence between populations (Lowe et al., 2005; Reed & Frankham, 2003). Together these genetic changes will have detrimental effects on population fitness and viability (Leimu et al., 2006) and influence the potential for a species to adapt to ongoing or future environmental changes (Jump et al., 2009; Lande & Shannon, 1996; Manel & Holderegger, 2013; Vilas et al., 2006).

The impact of fragmentation on a plant population can reduce pollen and seed dispersal, increasing genetic drift and inbreeding, while lowering diversity. Small populations have fewer potential mates, which can lead to increased bi‐parental inbreeding and even selfing. In time, this can lead to increased genetic load and inbreeding depression (Kramer et al., 2008). This increased genetic load can increase the risk of extinction if populations cannot withstand the consequences of inbreeding depression (Wallace, 2003). Increasing migration between small remnant populations, either through creation of corridors or movement of individuals, increases genetic diversity (Submitting author et al. in prep) and is used as a management tool for populations impacted by fragmentation (Pavlova et al., 2017; Whiteley et al., 2014). However, in some plant species, there is evidence that fragmentation can have a positive impact on gene flow (Breed et al., 2015; Matesanz et al., 2017), although it has been suggested that this paradox is the product of sampling individuals established pre‐fragmentation (Breed et al., 2013; Vranckx et al., 2011), rather than seedlings or younger individuals that arose post‐fragmentation (Breed, Marklund, et al., 2012; Breed et al., 2013; Breed, Stead, et al., 2012; Vranckx et al., 2011; Yates et al., 2007).

Successfully predicting the effects of anthropogenic habitat loss and fragmentation on genetic diversity and species resilience requires an integrated approach that considers genetics, demographics, isolation, and habitat quality across a species’ range (Lowe et al., 2005). To this end, we conducted a range‐wide genetic analysis that included assessment of population size, isolation, and habitat availability of the federally threatened orchid *Platanthera leucophaea* (Nuttall) Lindley (eastern prairie fringed orchid). We sampled populations spanning from critically small (*n* < 5) to large, robust population sizes (*n* > 1000). Our objectives were to (i) investigate range‐wide genetic patterns in *P. leucophaea* to determine if we can detect the underlying genetic structure of the species before fragmentation, (ii) determine the relationship between population size and genetic variation, and (iii) determine if changes in genetic parameters might indicate if populations impacted by fragmentation are heading toward extinction.

## 2 METHODS

### 2.1 Study species

The eastern prairie fringed orchid (*Platanthera leucophaea*) is a long‐lived, perennial, terrestrial orchid (Bowles, 1983, Figure 1) native to high‐quality wetland communities including wet prairies, sedge meadows, fens, and bogs of North America. The species was once found in contiguous wetland communities east of the Mississippi River, south of Ontario, and north of Kentucky. Historically, populations numbered in the thousands (Bowles, 1983; COSEWIC, 2003; Figure 2); however, due to habitat loss and fragmentation, the total number of populations has decreased by over 70% in the United States (USFWS, 1999). Remaining populations occur in small remnants with diminished numbers of individuals (from 1 to over 1000 individuals; Table1). As a consequence, *P. leucophaea* was federally listed as threatened in the United States in 1989 and federally listed as endangered in Canada in 2005 (COSEWIC, 2003). Today, there are 97 documented populations of *P. leucophaea* known to exist in the United States (Illinois, Indiana, Iowa, Maine, Michigan, Missouri, Ohio, Wisconsin (USFWS, 2016)) and 21 in Canada (Ontario, COSEWIC, 2003).

**FIGURE 1:**
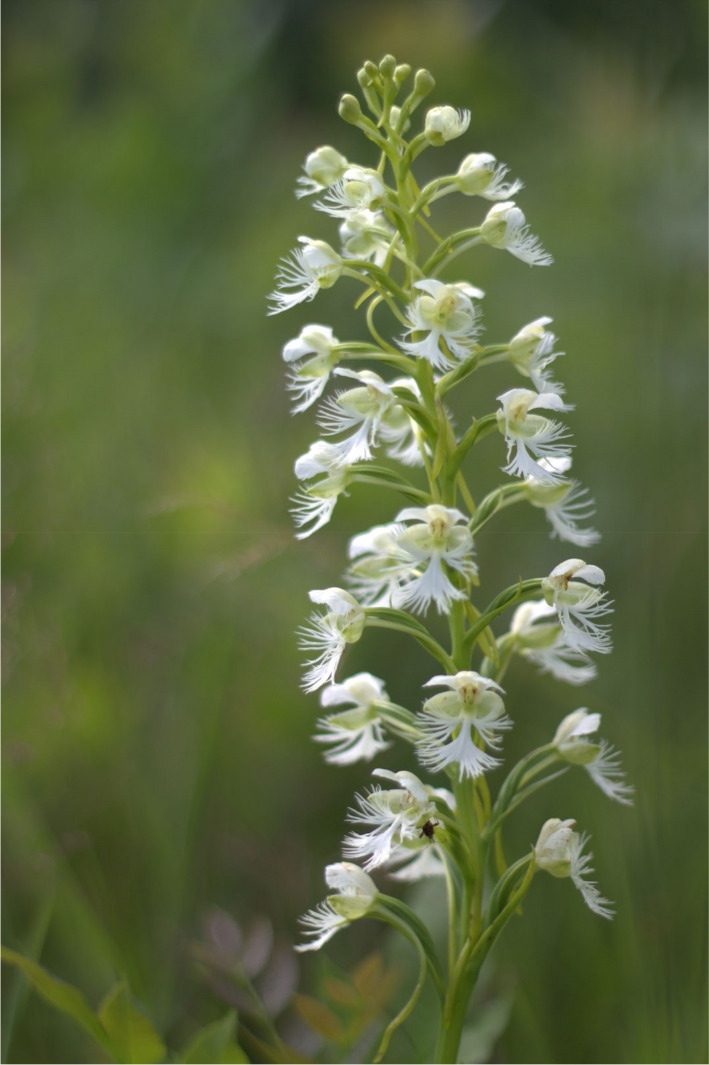
*Platanthera leucophaea*, the eastern prairie fringed orchid. Photo: Rachel Wells

**FIGURE 2:**
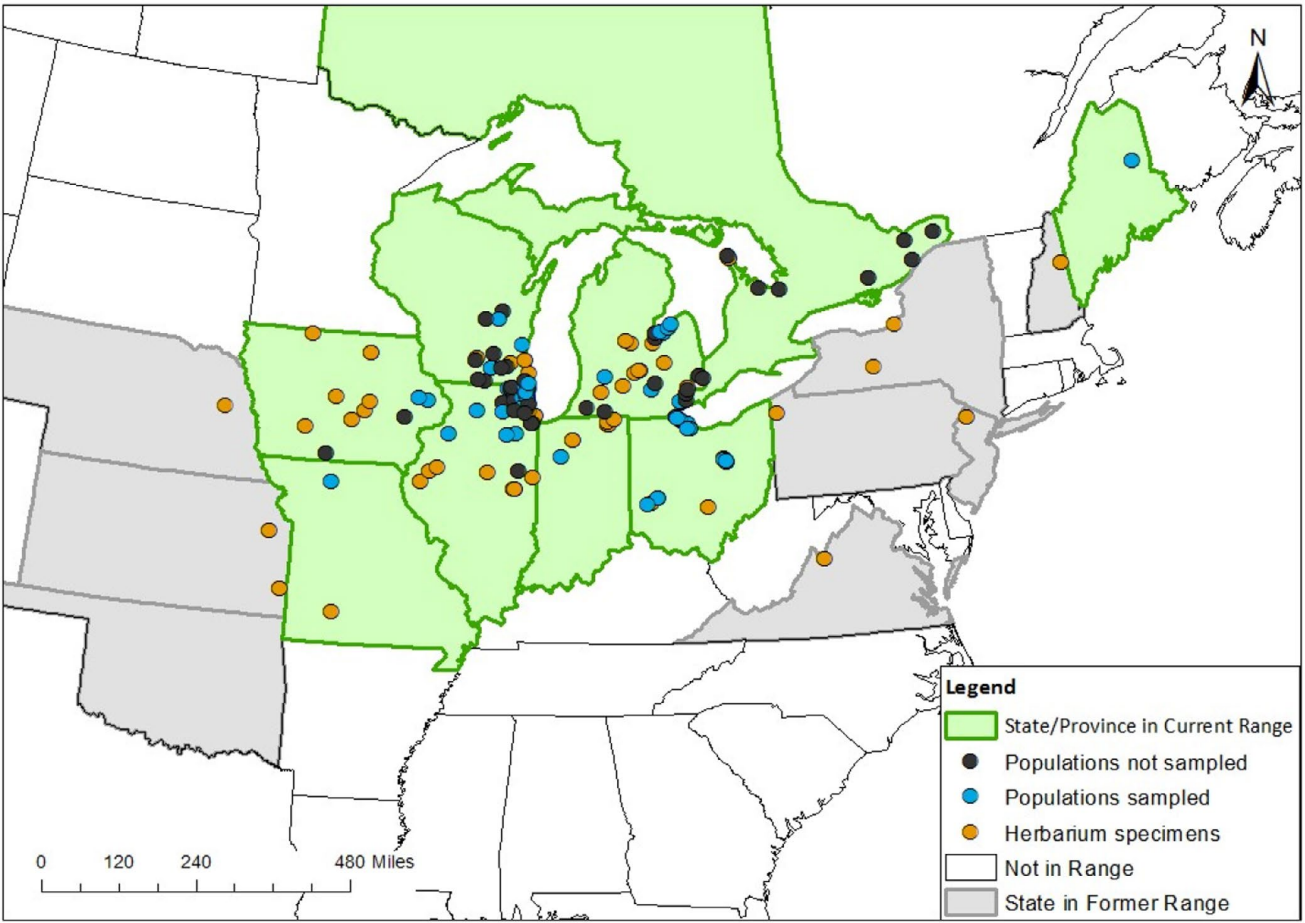
Present day and historic range map of *P. leucophaea* in North America, with populations sampled in blue, all other extant populations in black, and locations of herbarium specimens in orange

**TABLE 1:** Genetic data for 26 large and 10 critically small populations (*n* < 18) of *Platanthera leucophaea* that span the range of the species. Population metrics include name code (abbr.), state, latitude and longitude, sample size, and census size in 2015. The genetic measures calculated include effective population size (N_eff_), average number of alleles (Na), effective number of alleles (N_e_), genetic diversity (H_e_), inbreeding coefficient (F_is_), relatedness (*r*), average pairwise F_st_, and average pairwise G_st_. Contact the author for location information

Code	State	Samples size	Census size (2015)	Eff. Pop. Size (N_eff_)	# Alleles (Na)	# Eff. Alleles (Ne)	Genetic div. (H_e_)	Inbreeding (F_is_)	Relatedness (*r*)	Ave. Pairwise F_st_	Ave. Pairwise G_st_
MS	IA	30	75	38.40	7.63	4.82	0.74	0.12	0.04	0.08	0.08
BM	IA	30	600	56.80	9.00	6.15	0.79	0.11	0.04	0.09	0.09
CB	WI	29	75	58.30	7.88	5.03	0.67	0.07	0.03	0.08	0.08
FM	WI	30	63	44.80	7.63	4.39	0.74	0.11	0.04	0.07	0.07
KW	WI	30	100	62.60	9.00	6.11	0.77	0.15	0.02	0.06	0.07
UPA	WI	30	100	33.80	8.00	5.09	0.76	0.05	0.04	0.07	0.07
CH	WI	31	75	95.60	8.88	4.81	0.71	0.14	0.02	0.06	0.06
BS	IL	29	30	112.90	7.38	3.71	0.70	0.08	0.08	0.09	0.09
WT	IL	30	33	38.40	9.00	5.36	0.71	0.18	0.02	0.06	0.06
MC	IL	30	39	341.40	7.88	5.31	0.75	0.08	0.04	0.08	0.07
LY	IL	30	103	57.60	8.25	5.52	0.75	0.07	0.03	0.06	0.06
GC	IL	30	110	12.50	6.63	4.57	0.70	0.12	0.08	0.10	0.10
W	IL	30	347	43.70	9.13	5.35	0.72	0.13	0.02	0.07	0.07
SOM	IL	30	394	27.10	6.88	4.03	0.69	0.20	0.07	0.08	0.08
PM	MI	18	18	434.20	7.38	4.47	0.68	0.04	0.04	0.08	0.07
SB	MI	30	100	112.80	7.75	4.12	0.66	0.10	0.06	0.10	0.09
LCL	MI	31	38	40.50	6.75	3.43	0.67	0.07	0.13	0.15	0.13
MO	MO	30	57	107.10	7.38	4.08	0.69	0.08	0.08	0.10	0.09
YA	OH	23	23	183.60	7.38	4.36	0.70	0.01	0.06	0.08	0.08
MTZ	OH	25	25	82.20	7.75	4.88	0.74	0.07	0.06	0.09	0.08
PC	OH	28	334	Inf	8.38	5.34	0.72	0.06	0.03	0.06	0.06
NT	OH	29	135	46.50	8.50	5.58	0.76	0.08	0.03	0.06	0.06
YO	OH	29	1316	36.90	6.38	3.90	0.70	0.12	0.09	0.12	0.11
KB.83	OH	30	68	115.40	8.13	4.23	0.73	0.01	0.04	0.09	0.08
LD	OH	30	132	Inf	8.88	5.41	0.74	0.19	0.01	0.06	0.06
CC	OH	30	654	81.60	8.00	4.92	0.74	0.15	0.05	0.07	0.07
Critically small populations											
Long	IL	6	6	12.70	4.50	3.72	0.69	−0.34	0.10	0.10	0.08
IN	IN	5	5	9.70	4.50	3.68	0.68	−0.09	0.01	0.07	0.06
ME	ME	5	15	3.70	2.63	1.87	0.39	0.44	0.20	0.32	0.28
BG	MI	10	10	78.50	5.63	3.96	0.66	0.01	0.08	0.08	0.08
DC	MI	7	7	31.20	4.63	3.33	0.59	−0.04	0.13	0.22	0.18
SW	MI	13	158	25.60	5.38	3.58	0.66	0.14	0.07	0.10	0.10
MI	MI	7	7	11.70	4.00	2.83	0.60	−0.22	0.15	0.15	0.13
MDW	OH	13	15	22.50	6.88	4.49	0.72	0.09	0.03	0.07	0.07
KB.N	OH	6	8	63.80	3.38	2.49	0.52	−0.05	0.17	0.17	0.15
KB.S	OH	3	6	1.80	3.25	2.85	0.63	−0.47	0.09	0.07	0.06

*Platanthera leucophaea* can self‐pollinate but relies on pollinators to produce seed. The main pollinators are nocturnal flying hawkmoths in the family Sphingidae (Bowles, 1983; Cuthrell et al., 1999). *P. leucophaea* flowers produce dust‐like seed that is thought to be wind dispersed. Hence, despite the highly fragmented distribution of extant populations, long‐distance pollination by hawkmoths and seed dispersal by wind may facilitate some gene flow between populations. Germination requires mycorrhizae (Zettler & Piskin, 2011) before a protocorm is formed, which may stay dormant for several years depending on nutrients supplied by mycorrhizal fungi. Plants may take 2–13 years to reach reproductive maturity (USFWS, 2016).

### 2.2 Study sites

For this study, we selected 36 *P. leucophaea* populations that span its US range (Figure 2) and vary in population size, patch isolation, composition, and area. In states that the species is extant, we targeted populations that were historically the largest (based on available census sizes from the USFWS; Table S1). From each population, leaf tissue was haphazardly collected from 30 flowering individuals or all flowering individuals, if a population had less than 30 plants. One population (ME) had no flowering plants and only vegetative plants were sampled from this population. We collected 4–5 cm from the leaf tip of each plant, and dried the tissue in silica gel for later DNA extraction. We sampled 25 populations in 2015 and 3 in 2016. DNA extractions provided by Lisa Wallace (Wallace, 2002) were used to fill in sampling gaps in Michigan (one population, 1999) and Ohio (three populations in 1998). We were particularly interested in including those populations that are critically small (<50) and therefore most likely to be impacted by inbreeding and loss of diversity associated with an extinction vortex. One of the challenges of including these populations is that the sample sizes are below the number recommended for accurate genetic assessment (Hale et al., 2012). To address this, we generated a sampling effort curve to determine the minimum sample size needed to give equivalent results; similar to species accumulation curves used in ecology (Fisher et al., 1943).

### 2.3 Molecular data

To characterize the genetic structure of each population, genomic DNA was extracted using a CTAB extraction protocol (Doyle & Doyle, 1987). DNA quality was estimated using a NanoDrop 2000 spectrophotometer (Fisher Scientific). Thirty‐one microsatellite primers previously developed by Ross et al. (2013) for *Platanthera praeclara* were screened for genotyping of *P. leucophaea*. Of the 31 primers, 9 did not amplify in *P. leucophaea*, 10 produced monomorphic peaks and 12 produced consistent banding patterns. Twelve microsatellite loci were identified for genotyping *P. leucophaea*, which amplified and produced consistent and reliable banding patterns (PP05, PP07, PP09, PP10, PP16, PP19, PP22, PP23, PP24, PP27, PP30, and PP31; Ross et al., 2013). These were used for genotyping all individuals using fluorescently tagged forward primers (Sigma‐Proligo). PCR reactions were performed in a 10 µl reaction mixture containing the following: 3 µl DNA, 0.5 µl forward and reverse primers, 0.125 µl BSA and 0.875 µl H_2_0, and 5 µl PCR master mix 2x (Promega, Madison). PCR was run at 94°C for 5 min, then 35 cycles of 94°C for 40s, 60°C for 40s, 72°C for 3 min, and then a final extension at 72°C for 10 min for six primers (PP05, PP07, PP22, PP16, PP19, and PP27), while for the remaining primers (PP09, PP10, PP23, PP24, PP30, and PP31), the extension step was extended to 1 min. Genotypes were scored using a CEQ 8000 Genetic Analysis System and CEQ FRAGMENT ANALYSIS software (Beckman Coulter).

### 2.4 Population variables

Population size, patch composition, and degree of geographic isolation were measured or calculated for all populations in multiple ways. Using multiple measurements is particularly important for population size, as orchid numbers can fluctuate significantly from year to year depending on environmental and local biotic conditions (USFWS, 1999). As metrics for population size, we included: (1) a census of population size at time of leaf collection in 2015 and 2016; (2) a calculated median population size, as well as minimum and maximum size in populations from yearly censuses (2003–2015); and (3) the categorical census size determined by the USFWS in the recovery plan (0 ≤ 10, 1 = 10–25, 2 = 25–50, and 3 > 50; USFWS, 1999; Table S1).

The metrics for patch composition were calculated using a 1, 10, and 20km buffer around each population and tabulating the landcover‐type areas within, using classifications of the 2011 Gap Analysis Program (GAP), National Landcover Data. As the 1, 10, and 20km proportions were all equivalent, we ultimately focused on the 1 km buffered area. To identify the spectrum of preferred habitat types of this species, we generated a list of the natural landcover types which intersected each population centroid from across the sampling area. We assumed, given that the species was found within these landcover types, that they represent potentially suitable habitat (Table S2). Therefore, to calculate the total potential patch area within the 1km buffer around each population, we summed the area for all landcover types in which *P. leucophaea* was found. The remaining landcover types were then characterized as either natural, agricultural, or developed landcover types (Table S2). Natural area was defined as any landcover which was not developed, agricultural, or suitable habitat for *P. leucophaea*. Once all landcover types were classified, we summed the area to give us the total area of suitable habitat, natural area, agricultural, and development within the 1km buffer surrounding each population. Ultimately, the metrics associated with patch composition included (1) the USFWS ranked categorical habitat sizes (0 = <2.5 acres; 1 = 2.5<62.5 acres; 2 = 62.5<125 acres, and 3 = >125 acres; USFWS, 1999), (2) the amount of suitable habitat (patch size), (3) natural area, (4) agricultural land, and (5) total development.

The degree to which each population was isolated was determined using the average pairwise distance between all populations, average distance to the 5 and 10 nearest extant populations, and landscape resistance. The average Euclidian pairwise distance was calculated in SPAGeDi (Hardey & Vekemans, 2002) from the latitude and longitude of each population. The average nearest‐neighbor distances to the 5 and 10 closest neighbors were calculated in ArcGIS 10.3.1 using all known extant populations. Landscape resistance is a distance metric calculated using Circuitscape, which accounts for the variability in landscape types for movement between populations. This required categorizing all of the landcover types from the Multi‐Resolution Land Characteristics consortium, National Land Cover Database (NLCD), from 0 to 5 based on its suitability to pollinator movement or habitat suitability for colonization (Table S3). Circuitscape then tests multiple trajectories through the landscape and produces a relative metric of the distance between populations based on landscape suitability and determine the shortest distance with the least landscape resistance connecting two populations (Shah & McRae, 2008).

### 2.5 Statistical analysis

#### 2.5.1 Genetic parameters

We used 12 microsatellites to measure factors commonly impacted by fragmentation and associated with declining populations which included: (1) genetic diversity (Schlaepfer et al., 2018), (2) effective population size (England et al., 2010), (3) inbreeding levels within (Leimu et al., 2010; Schlaepfer et al., 2018), and (4) differentiation between populations (Miles et al., 2019). All primers were tested for departure from Hardy–Weinberg Equilibrium (HWE) at the locus, population, and global levels, using Genepop (Raymond & Rousset, 1995). The potential of null alleles and mis‐scoring was tested using exact tests in Micro‐Checker (Van Oosterhout et al., 2004). Genetic diversity was quantified using effective number of alleles per locus (*N_e_*), and expected heterozygosity (*H*_e_), which were calculated in GENALEX (Peakall & Smouse, 2006). Both of these metrics are less sensitive to differences in sample size than other metrics. To measure rates of inbreeding within a population, we calculated Weir and Cockerham’s (1984) estimates of Wright’s inbreeding coefficient (F_IS_) and Queller and Goodnight’s Relatedness (R) in GENALEX (Peakall & Smouse, 2006). We used the linkage‐disequilibrium method in NeEstimator V2.01 (Do et al., 2014) to measure the population size, *N_eff_*, as it has good precision for microsatellite data with limited sample sizes and populations with small effective sizes (100–200; Waples & Do, 2008). *N_eff_* is an important parameter because it is not always reflected in inbreeding and genetic diversity variables (Bazin et al., 2006; Raymond & Rousset, 1995) and is impacted by fragmentation (England et al., 2010). Finally, to measure genetic differentiation, we used Rousset’s linearized F_ST_ (F_ST_/(1e−F_ST_; Rousset, 1997) and G_ST_ which is equivalent to F_ST_ but more appropriate for microsatellites (Pons & Petit, 1996). The pairwise genetic and spatial distances were calculated in SPAGeDi (Hardey & Vekemans, 2002).

#### 2.5.2 Sample size

Since the sample sizes in 12 populations were below the accepted sampling size (*n* < 25) for attaining accurate population genetic metrics (Hale et al., 2012), we generated a sample effort curve for He and Fis, similar to that proposed by Bashalkhanov et al. (2009) to identify the minimum sample size which produces accurate estimates of genetic parameters. To achieve this, we subsampled all populations with sample sizes larger than 25 using stratified. R code (https://gist.github.com/mrdwab/6424112). We randomly sampled individuals from each population to generate populations that ranged in sample sizes from 2 individuals to 25 individuals and repeated 10 times for each sample size. We then calculated the expected heterozygosity using the R package StrataG.R (Archer et al., 2017) and calculated inbreeding using the R package adegenet (Jombart, 2008) for all subsamples. To determine the minimum sample size at which heterozygosity and inbreeding results plateaued, we plotted the mean standard error of all samples using ggplot2 (Wickham 2016; Supplemental 3).

#### 2.5.3 Range‐wide genetic structure

To determine if there were range‐wide patterns in genetic structure within *P. leucophaea*, we used the Bayesian clustering analysis software STRUCTURE v2.2 (Falush et al., 2007; Pritchard et al., 2000) to visualize population subdivision (number of genetic clusters, K) among our 36 study populations. This software uses individual multilocus genotypes to test for the presence of population structure without a priori assignment of individuals to populations by finding population groupings with the least possible disequilibrium using a Markov Chain Monte Carlo method. We carried out 40 independent runs per *K* using a burn‐in period of 10^5^ and collected data for 10^5^ iterations for *K* = 1–40. The minimum value of *K* that can explain the data was assessed using the rate of change in the log‐likelihood probability of data between corresponding *K* values (Δ*K*) as detailed in Evanno et al. (2005) using Structure Harvester (Earl & vonHoldt, 2012).

To identify isolation by distance, we compared pairwise genetic distances F_ST_ (F_ST_/(1−F_ST_); Rousset, 1997) and G_ST_ (Pons & Petit, 1996) against Euclidian geographic distance for populations with sample sizes greater than 18 using the Mantel test in GENALEX (Peakall & Smouse, 2006). To determine if other genetic variables varied by location, we tested for correlations of H_E_ and N_E_ (genetic diversity), F_IS_ and relatedness (inbreeding), and N_eff_ (effective population size) against latitude and longitude using the correlation coefficient panel for pairs function in R Statistical Software (R Core Team, 2016).

#### 2.5.4 Modeling populations and genetic parameters

##### Principal component analyses

We used the princcomp function to create a principal component analysis (PCA) summarizing all variables into a single metric that captures the spectrum of variation for that trait. All variables were first tested for normality and were log‐transformed when appropriate (including area of habitat, developed area, natural landcover, census size in 2015, and the minimum, maximum, and median population sizes across the available years of the USFWS censuses (IL and OH only)). For population size, we used five variables: census in 2015 (Census), median census size (MedCensus), minimum and maximum size (Min & MaxCensus), and the categorical census size (CatPopSize). For patch composition, we used four variables: percent suitable habitat, percent natural area, percent agriculture, and percent development. Finally, for patch isolation, we used four measurements: average pairwise distance (Distance), average distance to 5 and 10 nearest extant populations (NeN5, NeN10), and landscape resistance as calculated in Circuitscape (resistance). For each PCA, we used the get_pca_var function in the R package factoextra 1.0.6 (Kassambara & Mundt, 2020) to determine the amount of variation explained by each axis (Eigenvalue and proportion of variation explained) and the contribution of each metric in explaining the spread. The predict function in Vegan (Okansen et al., 2019) was used to extract the coordinates for each population along the PCA axis. To check for independence of our different model parameters, we used cor.test function in R to look for association between our PCA axes and latitude and longitude.

##### Models

To investigate whether population and landscape variables explain genetic parameters, we used linear models that included all of the extracted axes that cumulatively explained at least 80% of the variation for the population size, patch isolation, and patch composition PCAs (see Table 2 for list of each metric used), as well as latitude and longitude to account for geographic variation in these traits. The simplest model was selected through backward and forward elimination using the StepAIC function in R Statistical Software. The best model was compared for homoscedasticity and then tested against the null hypothesis using the ANOVA function. The genetic parameters tested in the model were H_e_ and N_e_ as measures of genetic diversity, F_is_ and relatedness as measures of inbreeding, and pairwise F_ST_ and G_ST_, which are measures of fixation index often used as a proxy for genetic distance between two populations. As some populations were too small to accurately reflect genetic metrics of diversity and inbreeding, we created two datasets, one that contained data from all populations and a second that was restricted to just larger populations (*n* > 18). In addition to genetic parameters, we were also interested in how population sizes vary with anthropogenic changes. Therefore, we modeled both census size and effective population size, N_e_, against the PCA axes calculated to estimate the impacts of anthropogenic change, patch isolation, and patch composition. Since effective population size is not always correlated with census size, we modeled this term with and without the size PCA axes. After running the models, we found that the two disjunct populations in Missouri and Maine were two‐ to fourfold further from any other populations, making them large geographic outliers that had a disproportionate impact on the model analyses. Since these were effectively isolated, and to get a more accurate representation of processes in the center of its range, we excluded these populations from the model analyses.

**TABLE 2:** Breakdown of PCA results for a) population size, b) patch area, and c) patch isolation, including eigenvalues, proportion of variance explained, and breakdown of percent explained by each variable used in the analysis

PCA	All data (36 populations)				Only larger sample size (*n* > 18; 26 populations)			
a) Population Size	pcaSize1	pcaSize2	pcaSize3	pcaSize4	pcaSize1	pcaSize2	pcaSize3	pcaSize4
Eigenvalue	3.23	1.11	0.32	0.26	2.36	1.50	0.59	0.47
Total Variance Explained	65%	22%	7%	5%	47%	30%	12%	10%
Breakdown by Parameter								
Census	24.05	1.55	15.03	57.46	24.59	2.80	31.75	39.09
MinCensus	6.97	68.17	0.54	0.03	6.75	54.31	1.03	0.10
MaxCensus	19.94	23.17	9.72	18.43	16.28	30.77	2.64	25.98
MedCensus	26.30	5.44	1.36	22.56	29.48	10.51	0.06	24.31
CatPopSize	22.74	1.67	73.35	1.53	22.90	1.61	64.52	10.53

b) Patch Area	pcaArea1	pcaArea2	pcaArea3	pcaArea4	pcaArea1	pcaArea2	pcaArea3	pcaArea4
Eigenvalue	1.75	1.08	0.79	0.37	1.71	1.11	0.79	0.39
Total Variance Explained	44%	27%	20%	10%	43%	28%	19%	10%
Breakdown by Parameter								
Habitat (patch size)	0.07	84.78	7.71	7.44	0.20	80.16	8.51	11.12
Develop	41.30	1.55	10.28	46.87	40.95	2.90	11.50	44.64
Natural	19.65	6.42	73.56	0.37	20.60	7.32	71.53	0.55
Agric	38.98	7.25	8.45	45.32	38.25	9.62	8.45	43.68

c) Patch Isolation	pcaIso1	pcaIso2	pcaIso3	pcaIso4	pcaIso1	pcaIso2	pcaIso3	pcaIso4
Eigenvalue	3.28	0.48	0.21	0.01	3.22	0.47	0.29	0.01
Total Variance Explained	70%	21%	8%	0%	81%	12%	7%	0%
Breakdown by Parameter								
NeN5	27.03	27.87	0.00	45.10	25.94	33.78	0.38	39.90
NeN10	30.70	14.66	1.53	53.11	28.85	13.03	1.18	56.94
Resistance	22.47	21.47	56.05	0.01	22.60	24.22	53.19	0.00
Distance	19.80	36.00	42.42	1.78	22.62	28.97	45.25	3.16

#### 2.5.5 Impact of small population sizes on genetic data predictions

To determine if the trends in genetic parameters associated with including the critically small populations is a product of sampling bias or decreasing population sizes, we randomly selected seven individuals (the average size of our critically small populations) from all populations sampled using the stratified.R package. From this subset of data, we recalculated the effective number of alleles per locus (N_e_), expected heterozygosity (H_e_), and Weir and Cockerham’s (1984) estimates of inbreeding in GENALEX (Peakall & Smouse, 2006). We repeated this 10 times to generate multiple measurements of each parameter. These 10 replicates of each population were combined into a single dataset. We then used linear models in R to compare genetic diversity values (H_e_ and N_e_) and inbreeding (F_is_) to the natural log of the average census size. This allowed us to look for trends that were less impacted by sampling bias.

## 3 RESULTS

### 3.1 Descriptive statistics of populations

Four of the 12 microsatellite primers were excluded (PP09, PP10, PP16, and PP19) from our analyses after testing for null alleles, departure from Hardy–Weinberg equilibrium (HWE), and mis‐scoring. The sample effort curve revealed that the minimum sample size needed for an accurate measurement of genetic diversity (H_e_) and inbreeding (F_is_) for our dataset was *n* = 18. For those populations (>18 individuals), the average number of alleles by population (N_a_) ranged from 6.3 to 9.1 (ave 7.9), the number of effective alleles (N_e_) ranged from 3.4 to 6.1 (ave 4.8), and expected heterozygosity (H_e_) ranged from 0.66 to 0.79 (ave of 0.66). For the 10 populations where sample sizes were below 18, the range of the number of alleles (N_a_) was from 2.6 to 6.8 (ave 4.5), the number of effective alleles (N_e_) ranged from 1.8 to 4.5 (ave 3.3), and expected heterozygosity (H_e_) ranged from 0.39 to 0.72 (ave of 0.61), all of which were at the lower end of the ranges seen. The range for Weir and Cockerham’s (1984) estimates of Wright’s F_is_ varied from 0.01 to 0.20 (F_is_ = 0.10), suggesting a moderate level of inbreeding across populations; however, populations less than 18 spanned a large extreme from highly outcrossed (F_is_ = −0.47) and highly inbred (F_is_ = 0.44). Although, the highest measured inbred population was located in Maine (F_is_ = 0.44), which is a geographically disjunct population and likely an outlier, the next highest value was in Michigan (F_is_ = 0.14).

### 3.2 Analysis of population genetic structure

Structure Harvester identified two genetic clusters (K) between our populations with a slight geographic gradient from west to east (Supplemental 1). Interestingly, the geographically disjunct population in Maine was comprised of almost equal amounts of both clusters. Pairwise genetic relatedness slightly increased with distance; the pairwise genetic distances (F_st_) for population pairs greater than 18 individuals ranged from low (0.01) to relatively high (0.16) with a low average of 0.06 (Supplemental 2). Nei’s D, pairwise genetic distance, followed a similar trend, increasing slightly with distance between populations.

### 3.3 Principal component analyses

**FIGURE 3:**
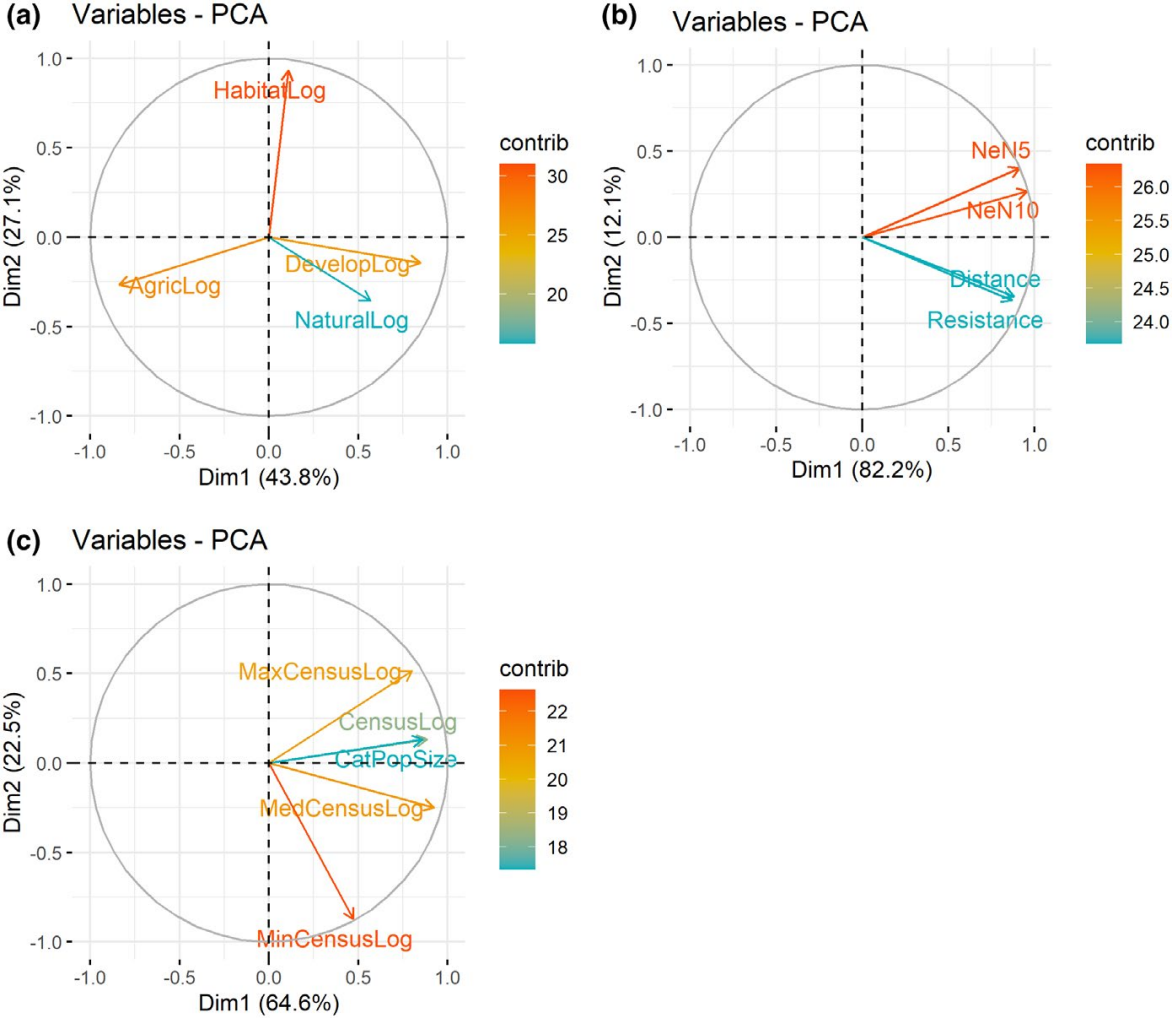
PCA variables and their contributions to the (a) habitat PCA, (b) isolation PCA, and (c) size PCA for all *Platanthera leucophaea* populations sampled

Differences in patch composition of all populations was 100% explained by the first four PCA axes (Table 2). The first axis (pcaArea1), which explained 43% of the variation, described the degree of development within the patch, being positively associated with the log area of urban development (41%) and negatively associated with the log area of agricultural development (38%). The second axis (pcaArea2), which explained 28% of the total variation, showed a strong association with total area of preferred habitat types (80%; Table 2; Figure 3). The final two axes explained 19% and 10% of the remaining variance, respectively (Table 2). For populations with >18 individuals (large populations), the first two axes explained most of the variation (71%; Table 2).

Differences in patch isolation between only the large populations was 100% explained by the first four axes, although the first axis explained 89% of that variation. Each metric of fragmentation (landscape resistance, average pairwise distance to 5 nearest and 10 nearest extant populations, and geographic distance) contributed equally to the variation along the first axis (Table 2). PCA axis 1 explained 81% of the differences in patch isolation between all populations with equal contributions from all four metrics (Table 2; Figure 3).

To quantify population size, we used four variables (census in 2015, median census size, minimum and maximum census size, and the categorical population size). Differences between population sizes were 98% explained by the first four PCA axes using four measurements of population sizes (census in 2015, median census size, minimum and maximum census size, and categorical population size), with 47% of the variation explained by census 2015, median census size, and categorical population size along axis 1 (pcaSize1). The second axis explained an additional 30% of the variation and was explained by the minimum census size. The remaining two axes explained 12% and 10% of the variation (Table 2). For all populations, the first axis explained 65% of the variation and was equally influenced by all metrics except minimum census size. By contrast, the second axis explained an additional 22%, which was mainly explained by minimum census size (68%; Table 2; Figure 3).

### 3.4 Correlations between model parameters

Before we ran the models, we tested all PCA axes for evidence of correlation between axes, as well as between latitude and longitude. Most pairs of axes showed no significant correlations with each other. An exception was patch isolation (pcaIso1), which was significantly negatively correlated with development (pcaArea1), and this was regardless of whether we used the dataset that includes all populations (*r* = −0.40, *t*_33_ = −2.5, *p* = .02) or only those with larger sample sizes (*r* = −0.40, *t*_25_ = 2.18, *p* = .04). And, patch isolation (pcaIso1) was also significantly negatively correlated with patch area (pcaArea2) regardless of whether we used the dataset that include all populations (*r* = −0.52, t_33_ = −3.5, *p* = .001) or only those with larger sample sizes (*r* = −0.57, *t*_25_ = 3.45, *p* = .02). This suggests that for our dataset we were unable to parse out completely the impacts of patch size (pcaArea2) and development (pcaArea1) from the degree of patch isolation. Interestingly, in this data, increasing isolation was associated with decreased development and larger population sizes. To investigate if there was a geographic pattern to the fragmentation parameters, we tested for correlations with longitude and latitude. We found no correlations between longitude and size (pcaSize1 or pcaSize2), but there was a significant positive relationship with patch size (pcaArea2) for both the complete dataset with all populations (pcaArea2, *r* = 0.58, *t* = 4.13, *p* = .0002) and the dataset restricted to only larger populations (pcaArea2, *r* = 0.62, *t* = 3.95, *p* = .0005), suggesting that patch areas to the east are larger. Similarly, the significant negative correlations between longitude and patch isolation (all pops, *r* = 0.34, *t* = −2.13, *p* = .04; and large only, *r* = 0.44, *t* = −2.48, *p* = .02) suggest most populations to the eastern edge of the range are also less isolated. Additionally, there was a significant negative relationship between latitude and patch isolation (all pops, *r* = −0.47, *t* = −3.10, *p* = .004; and large only, *r* = −0.50 *t* = −2.93, *p* = .007), suggesting southern populations are less isolated. There were no correlations between latitude and patch size (pcaArea2), development (pcaArea1) or size (pcaSize1), but there was a significant positive correlation with the second PCA axis of size (pcaSize2) and latitude for both datasets (all pops, *r* = −0.38, *t* = −2.05, *p* = .05; and large pops only, *r* = 0.42, *t* = 2.66, *p* = .01). Although this axis only explained 6–12% of the variation for size, it is strongly negatively influenced by minimum census size. This might suggest that northern populations have recorded some of the smallest census sizes.

### 3.5 The relationship between genetic parameters and population metrics

**FIGURE 4:**
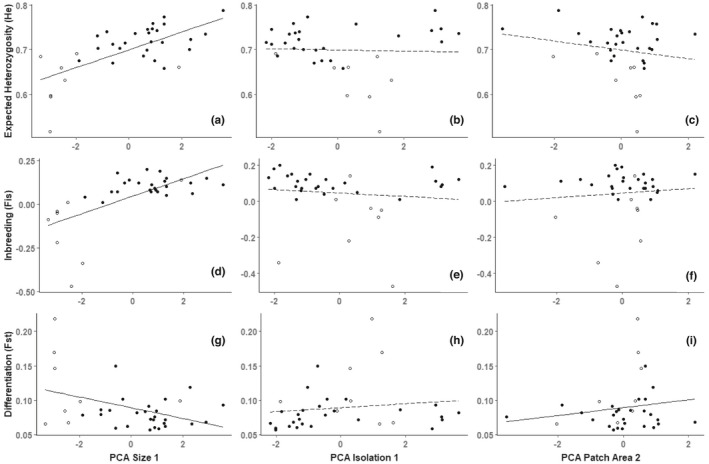
Expected heterozygosity (He) (a–c), inbreeding (F_IS_) (d–f), and differentiation (F_ST_) (g– i) by PCA size axis 1 (a, d & g), PCA isolation axis 1 (b, e, & h), and PCA patch area axis 2 (c, f, & i) for all populations of *Platanthera leucophaea*. Large populations, *n* > 18, are displayed with black circles, and small populations are hollow circles. Linear model (all populations), (a): *p* ≤ .001; (b): *p* = .13; (c): *p* = *NS*; (d): *p *≤ 0.001; (e): *p* = *NS*; (f): *p* = *NS*; (g): *p* = .009; (h): *p* = .09; and (i): *p* = .04). Solid line, *p* ≤ 0.05, dashed line, *p* > .05, *p* = NS, not supported in linear model

For the models that included all populations, the variables that best explained the effective number of alleles (Ne; *R* ^2^ = 0.53, *R* ^2^_adj_ = 0.49, *F*_3,30_ = 11.55, *p* < .001) included population size (pcaSize 1, *p* < .001 & pcaSize 2, *p* = .15) and longitude (*p* = .03 & p_large only_ = 0.17; Table 3). Similarly, the best model for expected heterozygosity (*R* ^2^ = 0.60, *R* ^2^_adj_ = 0.53, *F*_5,29_ = 8.5 *p* < .001) also retained size (pcaSize1, *p* < .001) and longitude (*p* = .01), but also included patch isolation (pcaIso1, *p* = .13), amount of development (pcaArea1, *p* = .19), and latitude (*p* = .04). When we used the more restricted dataset the best model for effective number of alleles (Ne) was less supported (*R* ^2^ = 0.23, *R* ^2^_adj_ = 0.16, *F*_2,23_ = 3.5, *p* = .05) but still included both population size (pcaSize 1, *p* = .07) and longitude (*p* = .17). While with expected heterozygosity, the best model (*R* ^2^ = 0.46, *R* ^2^_adj_ = 0.36, *F*_3,22_ = 4.59, *p* = .01) also retained size (pcaSize1, *p* = .16) and longitude (*p* = .005), but it also included latitude (*p* = .01). In all scenarios, the genetic diversity metrics decreased with population size (Figure 4) and surprisingly longitude (from east to west), which is likely associated with greater development in the northwest of the range. The models that best explained levels of inbreeding (F_is_) in the full dataset (*R* ^2^ = 0.41, *R* ^2^_adj_ = 0.40, *F*_1,32_ = 22.5 *p* < .001) and the large population‐only dataset (*R* ^2^ = 0.38, *R* ^2^_adj_ = 0.26, *F*_4,21_ = 3.2, *p* = .03) both retained population size a predictor (pcaSize1, *p* < .001 and *p* = .14, respectively), and the large populations dataset also retained development (pcaArea1 *p* = .04) and longitude (Longitude *p* = .02) as strongest predictors. By contrast, the models that tested for Relatedness (R) using only large populations did not retain any variables, but when we included all populations, the best model (*R* ^2^ = 0.32, *R* ^2^_adj_ = 0.29, *F*_2,31_ = 7.62, *p* = .001) was negatively correlated with population size (pcaSize1, *p* = .001) and patch area (pcaArea2, *p* = .05). The models for degree of genetic differentiation (F_st_, G_st_) when using only large populations did not retain any variables; however, when we included all populations, the models for both F_st_ (*R* ^2^ = 0.28, *R* ^2^_adj_ = 0.21, *F*_3,30_ = 3.97, *p* = .02) and Gst (*R* ^2^ = 0.26, *R* ^2^_adj_ = 0.18, *F*_3,30_ = 3.55, *p* = .03) retained population size (pcaSize1, *p* = .009 and *p* = .02, respectively), patch isolation (pcaIso1, *p* = .09 and *p* = .08, respectively), and patch area (pcaArea2, *p* = .04 and *p* = .04, respectively). In all cases, the degree of differentiation increased with decreasing population size (Figure 4), decreasing patch area, and increased patch isolation (Table 3).

**TABLE 3:** Results of the linear models (F statistic (*F*), degrees of freedom (df), *p* value (*p*) and regression fit (both multiple and adjusted R squared)) comparing population characteristics, including population size (pcaSize1 and pcaSize2), patch isolation (pcaIso1) and patch area (pcaArea1, pcaArea2, and pcaArea3), and latitude and longitude against the following genetic parameters: effective population size (N_eff_), genetic diversity (N_a_, N_e_, and H_e_), inbreeding (F_is_ and relatedness), and differentiation (F_st_ and G_st_). We also tested how population size is dependent on patch isolation, patch area, latitude, and longitude. Models were repeated with (a) all data (36 populations), and (b) restricted to only larger (sample size *(n)* > 18) sample sizes (26 populations)

Variable	*F*	*df*	*p*	Multiple *R* ^2^/ Adjusted*R* ^2^	pcaSize1	pcaSize2	pcaIso1	pcaArea1	pcaArea2	Lat	Long
(a) All data (36 populations)											
Na	*29.67*	*1,32*	<.001	0.48/0.47	<0.001	*‐*	*‐*	*‐*	*‐*	*‐*	*‐*
Ne	*11.55*	*3,30*	<.001	0.53/0.49	<0.001	*0.15*	*‐*	*‐*	*‐*		*0.03*
He	*8.53*	*5,28*	<.001	0.60/0.53	<0.001	*‐*	*0.13*	*0.19*	*‐*	*0.04*	*0.003*
Fis	22.55	1,32	<.001	0.41/0.40	<0.001	‐	‐	‐	‐	‐	
Selfing	NS	‐	‐	‐	‐	‐	‐	‐	‐	‐	‐
Relatedness	*7.62*	*2,31*	.*002*	0.32/0.29	*0.001*	*‐*	*‐*	‐	*0.05*	*‐*	*‐*
Fst	*3.97*	*3,30*	.*02*	0.28/0.21	*0.009*	*‐*	*0.09*	*‐*	*0.04*		
Gst	*3.55*	*3,30*	.*03*	0.26/0.18	*0.02*	*‐*	*0.08*	*‐*	*0.04*	*‐*	*‐*
Census Size	3.04	2,31	.07	0.16/0.10	NA	NA	‐	‐	0.02	‐	0.07
Effective Population Size	3.35	1,32	.08	0.09/0.06	NA	NA	‐	0.08	‐	‐	‐
(b) Large sample size (*n* > 18; 26 populations)											
Na	NS	‐	‐	‐	‐	‐	‐	‐	‐	‐	‐
Ne	3.50	2,23	.05	0.23/0.16	0.07	‐	‐	‐	‐	‐	0.17
He	4.59	4,21	.01	0.46/0.36	0.16	‐	‐	0.12	‐	0.01	0.005
Fis	3.2	4,21	.03	0.38/0.26	0.14	‐		0.04	‐	0.11	0.02
Selfing	4.57	3,22	.01	0.38/0.30	0.01	‐	0.01	‐	‐	0.07	‐
Relatedness	NS	‐	‐	‐	‐	‐	‐	‐	‐	‐	‐
Fst	NS	‐	‐	‐	‐	‐	‐	‐	‐	‐	‐
Gst	NS	‐	‐	‐	‐	‐	‐	‐	‐	‐	‐
Census Size	1.99	1,24	.17	0.07/0.03	NA	NA	‐	‐	0.17	‐	*‐*
Effective Population Size	3.29	2,23	.05	0.22/0.15	NA	NA	‐	‐	0.16	‐	0.01

The models for census size using only large populations (*R* ^2^ = 0.07, *R* ^2^_adj_ = 0.03 *F*_1,24_ = 3.29, *p* = .05) retained only patch area (pcaArea2, *p* = .17), while the model using all population (*R* ^2^ = 0.16, *R* ^2^_adj_ = 0.10, *F*_2,31_ = 3.0, *p* = .07) retained both patch area (pcaArea2, *p* = .02) and longitude (*p* = .07). For effective population, only the models using large population (*R* ^2^ = 0.22, *R* ^2^_adj_ = 0.15 *F*_2,23_ = 3.28 *p* = .05) retained patch area (pcaArea2, *p* = .16), while models using all populations (*R* ^2^ = 0.09, *R* ^2^_adj_ = 0.07, *F*_1,32_ = 3.4 *p* = .07) retained development (pcaArea1, *p* = .08) and longitude (*p* = .01). This suggests that patch area is the best predictor of population size, with larger patches having larger populations. The association of size and longitude likely reflects the higher number of small populations to northeast of the range (Table 3).

### 3.6 Impact of small sample size on models

For critically small populations, sample size can make it difficult to draw conclusions when comparing genetic data from populations with larger samples sizes (>30). To correct for differences in sample sizes, we subsampled all populations to generate populations with seven randomly selected individuals (hereafter, all populations). We found that the adjusted average values of N_E_, H_E_, and F_IS_ measured on only seven individuals decreased compared to the original value (Supplemental 4). The magnitude of change somewhat explains the difference in the range of values seen between the 27 larger population (>18) and 10 smaller populations (≤18). For genetic diversity metrics, N_E_ and H_E_, the average standard deviation across the 10 replicates was within the range of the difference seen between subsampled and complete datasets (st.dev = 0.61 and 0.06, respectively). By contrast, the average standard deviation between the complete and subsampled data for inbreeding (F_IS_) was much greater (*SD* = 0.15). The large range of variation in the inbreeding coefficient likely reflected that populations comprised of only seven individuals will vary more in the ratio of related and unrelated individuals. Although we found negative F_IS_ values within subsampled datasets for most populations, all replicates of the critically small populations’ subsampled datasets had low‐to‐negative inbreeding values suggesting that for these populations this result is not a product of chance or small sample size alone.

Despite a clear downward trend in absolute values in all three metrics for the subsampled datasets, we found that there was a significant negative relationship between the log of census size and both measures of genetic diversity (N_E_, *F*_1,368_ = 113.6, *p* < .001; and H_E_, *F*_1,368_ = 98.36, *p* < .001). Interestingly, F_IS_ of the subsampled populations was also negatively influenced by population size based on the 2015 census size (*F*_1,368_ = 43.32, *p* < .001), like the results from the complete population samples (Figure S4). Hence, although the absolute values from the subsampled data were lower, the negative trend persisted (Figure S4). We interpret this to mean that the values for these smaller populations are a product of both sampling size bias and biological changes associated with small population sizes.

## 4 DISCUSSION

In this range‐wide study of the federally listed eastern prairie fringed orchid, *Platanthera leucophaea*, we found low levels of differentiation between populations, and moderate levels of inbreeding and genetic diversity within populations. There was a weak positive correlation between genetic and geographic distance from the eastern‐to‐western end of the orchid range (approx. 2000 km). Southern populations had slightly more genetic diversity than the northern populations. The low genetic differentiation and weak isolation by distance suggest historically high levels of gene flow between *P. leucophaea* populations. Over the last 100 years, human development and agricultural encroachment have fragmented *P. leucophaea* habitat, and as a consequence many populations have gone extinct and over 70% of the remaining populations have been reduced in size (USFWS, 1999). Of the many changes due to fragmentation, population size was consistently the best predictor of all genetic factors. For other parameters, the degree of genetic differentiation was shown to increase with patch area and isolation, while increasing patch isolation and development were associated with lower diversity and increased inbreeding. These relationships were particularly evident for the critically small populations (<18 individuals remaining). Therefore, a good indicator of potential genetic issues, when fragmentation occurs, is a drop in population size associated with less habitat area, especially when in an urban matrix (Miles et al., 2019). Given the yearly fluctuations in population sizes in orchid species, consistent monitoring is an important method to assess for extinction risk (Mace & Purvis, 2008).

Across the extant range of the species, we found relatively high levels of genetic diversity in many populations. The high diversity seen across the range is comparable to high levels of genetic variation found by previous studies (Havens & Alex Buerkle, 1999; Wallace, 2002). Many of the metrics used for diversity showed a slight trend with latitude and longitude and suggest that there is higher diversity to the southwest and lower diversity to the northeast. Considering the likely post‐glacial migration routes of *P. leucophaea*, the higher genetic diversity in the southwest could also be a product of it representing historic refugia (Hampe & Petit, 2005), which is supported by the lower diversity in the northeast of its range (Paul et al., 2013). A recent study in a sister species, *P. praeclara* (Ross & Travers, 2016) observed a similar range of diversity to *P. leucophaea* but higher number of alleles per locus, which might be a product of ascertainment bias, since these markers were developed in *P. praeclara*.

Despite the fragmentation, we found that larger populations show low levels of differentiation and low to moderate inbreeding. This is not surprising given that this orchid was historically widespread, occurred in large populations (USFWS, 1999), and is pollinated by long‐distance fliers (Bawa, 1990; Haber & Frankie, 1989; Linhart & Mendenhall, 1977; Skogen et al., 2019). These results differ somewhat from previous genetic studies, but this difference is likely driven by their focus on smaller populations, which also show elevated differentiation values compared to the larger populations in our study (Havens & Alex Buerkle, 1999; Paul et al., 2013; Wallace, 2002). Studies in the closely related *P*. *praeclara*, with similar hawkmoth pollinated flowers and wind‐dispersed seed, found comparable levels of genetic differentiation to this study (Pleasants & Klier, 1995; Ross & Travers, 2016). Our findings are also consistent with average levels of genetic differentiation found in other species of Orchidaceae (Machon et al., 2003; Phillips et al., 2012). Although *P. leucophaea* is now rare and populations are highly fragmented, the observed low F_st_ across the range is consistent with what is expected for a once historically widespread perennial species with wind‐dispersed seeds and long‐distance pollinators (Duminil et al., 2007; Loveless & Hamrick, 1984).

Population size consistently had the strongest relationship with changes in genetic diversity and inbreeding within a population. Past studies in *P. leucophaea* did not find that population size, measured as either harmonic mean (Wallace, 2002) or census size of the year sampled (Havens & Alex Buerkle, 1999), was correlated with genetic variation. This is likely an issue of sampling a limited number of populations and the high variability that most measurements for population size will produce. Orchid populations can fluctuate dramatically from year to year (Shefferson et al., 2019), making it difficult to produce a measurement that accurately reflects the population status. In our study, we overcame these issues by increasing the number of populations and using a PCA to incorporate different measures of population size (census in 2015, median, and the categorical population size) and fluctuations (census minimum and maximum size). Given that smaller populations are more susceptible to genetic drift and elevated inbreeding, the patterns we saw follow expectation, especially when considering the critically small populations. Previous literature reviews have also found a similar relationship to the size and population genetic diversity regardless if the species are rare or not (Honnay & Jacquemyn, 2007a). More importantly, *P. leucophaea* population size was positively correlated with a loss in fitness as well as genetic diversity (Leimu et al., 2006), supporting the concerns that these factors interact to accelerate the extinction vortex.

Surprisingly, we found that as *P. leucophaea* population size decreased, inbreeding also decreased, contrary to previous reviews which found a negative or no correlation (Angeloni et al., 2011). The positive relationship in our populations is likely driven by two biological phenomena: 1) that large populations of *P. leucophaea* maintain moderate‐to‐low levels of inbreeding, and 2) most of the populations with critically low numbers had negative inbreeding coefficients. Previous studies have reported similarly high levels of inbreeding for larger populations of *P. leucophaea* (Paul et al., 2013; Wallace, 2002) and *P*. *praeclara* (Ross & Travers, 2016). High inbreeding values in orchids are not unexpected. Orchids package their pollen into pollinaria, which ensures greater transport efficiency and maximizes the number of pollen grains reaching the stigma to assure high seed set (Harder, 2000; S. D. Johnson et al., 2005). However, the percentage of pollinaria that reach the stigma of another flower is low (<5% in Disa (S. D. Johnson et al., 2005)); roughly half of individuals rely on self‐pollination (Nilsson et al., 2009; Luyt & Johnson, 2001; Tremblay, 1994; Peakall, 1989; Salguero‐faria & Ackerman, 1999; Nilsson, 1992; S. D. Johnson et al., 2005). The other reason for this positive relationship is the negative inbreeding values in critically small populations, which suggests that mating is disassortative (i.e., only between unrelated individuals; Rasmussen, 1979; Stoeckel et al., 2006). Several different mechanisms have been proposed to explain this phenomenon, especially in critically small populations. The first is mate limitation, where species that cannot self will favor selection for the rarer allele (or sex; Hoebee et al., 2007; Sujii et al., 2015). Similarly, in species that are clonal (Halkett et al., 2005) or capable of unisexual reproduction (Johnson & Jonathan Shaw, 2015), there can be strong linkage disequilibrium generating large negative inbreeding values as the genetic lines diverge. Given that *P. leucophaea* is self‐compatible and non‐clonal, we do not think this is the situation in our study. The other possibility is that selection is favoring heterozygosity (heterosis). Although heterosis is beneficial in some systems (Stilwell et al., 2003; Oakley et al., 2015; Oakley et al., 2019; Bensch et al., 2006; W. H. Lowe et al., 2017), the fact that we are only seeing this in the smallest populations suggests that selection against homozygosity, associated with elevated inbreeding depression, is more likely (Aguilar et al., 2019; Angeloni et al., 2011; Charlesworth & Charlesworth, 1987).

The reduction in fitness associated with elevated homozygosity, known as inbreeding depression, is commonly seen in orchids (Juillet et al., 2007; Ortiz‐Barney & Ackerman, 1999; Sletvold et al., 2012). Several studies have shown a reduced seed production in selfed plants, including orchids (Sletvold et al., 2012), the genus *Platanthera* (Gregg, 1990; Nilsson, 1983; Patt et al., 1989; Travers et al., 2018), and more specifically this species (Wallace, 2003). However, inbreeding depression can also express itself in other demographic life stages including germination, seedling performance, and survival (Juillet et al., 2007; Sletvold et al., 2012). It is not uncommon for inbreeding coefficients to decrease as seeds transition to seedlings and then into adults, when most genetic sampling occurs (Aguilar et al., 2019; Cabin et al., 1998; Del Castillo, 1994; Honnay et al., 2008; Oostermeijer et al., 1995; Richards, 2000; Tonsor et al., 1993). The one critically small population that had elevated inbreeding levels was Maine, but it is also the only population where all sampled individuals were juvenile, supporting the hypothesis of higher inbreeding values in earlier life stages (Tonsor et al., 1993). Thus, we hypothesize that the negative inbreeding coefficient observed in our smaller populations is a result of a limited number of individuals surviving to flowering due to loss of individuals to inbreeding depression in early stages. This has been observed in other orchid species (Juárez et al., 2011) and will likely threaten the future viability of these populations (Spielman et al., 2004), emphasizing the need for conservation efforts within small *P. leucophaea* populations.

Additionally, we found support that important predictors of change to inbreeding, genetic differentiation, and diversity were patch isolation and the degree of urban development. The strength of these relationships was weaker than the population size, although this is likely because the three predictors were highly correlated in opposite directions. Many of the smaller populations were surrounded by greater urbanization but were also closer together and therefore less isolated. Thus, it was harder to untangle the impact of patch isolation versus development and patch area on our populations. This was reflected in our model of census size, which was associated with longitude and patch area, where the smaller populations on the western edge of the range had less available natural habitat. Similarly, the effective population size was lowest where urban development was the highest. Populations which showed some of the largest drops in population size over the censused years (minimum census size) were those populations to the north. Together these analyses show that populations in the Chicago Metropolitan area, at the northwestern edge of the range, have some of the smallest population sizes. The association between urbanization and population size suggests these populations are at high risk of going extinct.

The lack of relationship between genetic parameters and patch isolation suggests that it has less of an impact in this species. However, the lack of association in our data may be driven by many of our small populations occurring in close proximity, hence preventing us from disentangling the impacts of population size from patch isolation. However, as we also saw a lack of isolation by distance and weak differentiation across the range, this is likely a legacy of high rates of gene flow, at least in the past. This lack of differentiation is surprising, due to a long history of fragmentation and short generation times, but has been seen in other long‐distance pollinated species (Breed et al., 2013; Skogen et al., 2019). Long‐distance gene flow events, although commonly thought to be rare and difficult to document, are important for maintaining diversity in many systems. Hawkmoths in particular are of interest due to the fact that unlike bats and birds, which travel large distances but typically return to a nesting or roosting site daily, they are long‐distance flyers that migrate and are not home‐site specific. For these reasons, hawkmoths are expected to contribute to long‐distance gene flow despite multiple factors that may impede gene flow of *P. leucophaea* populations in modern fragmented landscapes (Aguilar et al., 2019).

*Platanthera leucophaea* has life history traits that are often associated with successful range edge expansion into newly hospitable habitat (long‐distance dispersal, highly mobile pollinators, and self‐compatibility; Parmesan, 2006). However, the loss of available habitat and reduction in population sizes will likely have long‐term consequences for the species. We found that a number of populations are showing signs of genetic decline, with evidence that populations are suffering from inbreeding depression and loss of genetic diversity. This was most evident in urban areas where the smaller populations have restricted patch area. Anthropogenic changes in patch area are directly related to population size changes, and we found that population size was a good indicator of genetic changes to populations of this orchid. Therefore, monitoring of these populations should continue to be prioritized in order to avoid population extinction due to genetic decline. More specifically, we note that populations with less than 15 flowering individuals are of highest concern and may lead to a demographic bottleneck if left unmanaged. Successive years of low census size may create a smaller “effective population size” and can therefore have serious genetic consequences. For some populations, which are showing signs of genetic decline, the augmentation of these populations, either through seed or pollen addition, is likely warranted. As many of these small populations have small neighboring populations, it is possible to conduct genetic augmentation with low risk of outbreeding depression, especially given the low differentiation recorded in this species (Amos et al., 2002; Frankham et al., 2011; Ralls et al., 2018).

## CONFLICTS OF INTEREST

The authors do not have any conflicts of interest.

## AUTHOR CONTRIBUTION

**Claire Ellwanger:** Conceptualization (equal); Formal analysis (equal); Investigation (equal); Writing – original draft (equal); Writing – review & editing (equal). **Laura Steger:** Investigation (equal); Writing – review & editing (equal). **Cathy Pollack:** Conceptualization (equal); Writing – review & editing (equal). **Rachel Wells:** Investigation (equal); Writing – review & editing (equal). **Jeremie Fant:** Conceptualization (equal); Formal analysis (equal); Writing – original draft (equal); Writing – review & editing (equal).

## Supporting information

Supplementary Material

## Data Availability

Genetic and landscape data are available on Dryad: DOI (https://doi.org/10.5061/dryad.j0zpc86fk).

## References

[ece38578-bib-0001] Aguilar, R. , Ashworth, L. , Galetto, L. , & Aizen, M. A. (2006). Plant reproductive susceptibility to habitat fragmentation: Review and synthesis through a meta‐analysis. Ecology Letters, 9(8), 968–980. 10.1111/j.1461-0248.2006.00927.x 16913941

[ece38578-bib-0002] Aguilar, R. , Cristóbal‐Pérez, E. J. , Balvino‐Olvera, F. J. , de Jesús, M. , Aguilar‐Aguilar, N.‐A. , Ashworth, L. , Lobo, J. A. , Martén‐Rodríguez, S. , Fuchs, E. J. , Sanchez‐Montoya, G. , Bernardello, G. , & Quesada, M. (2019). Habitat fragmentation reduces plant progeny quality: A global synthesis. Ecology Letters, 22(7), 1163–1173. 10.1111/ele.13272 31087604

[ece38578-bib-0003] Aguilar, R. , Quesada, M. , Ashworth, L. , Herrerias‐Diego, Y. , & Lobo, J. (2008). Genetic consequences of habitat fragmentation in plant populations: susceptible signals in plant traits and methodological approaches. Molecular Ecology, 17(24), 5177–5188. 10.1111/j.1365-294X.2008.03971.x 19120995

[ece38578-bib-0004] Amos, W. , Balmford, A. , Lewis, J. , Carstens, M. , Xia, C. , Keselowsky, B. , Brook, B. W. et al (2002). M Dürr ‐ Raubgut Berliner Biblioth ‐ TOC.Pdf. Oecologia, 172(1), 18–19.

[ece38578-bib-0005] Angeloni, F. , Joop Ouborg, N. , & Leimu, R. (2011). Meta‐analysis on the association of population size and life history with inbreeding Depression in Plants. Biological Conservation, 144(1), 35–43. 10.1016/j.biocon.2010.08.016

[ece38578-bib-0006] Archer, F. I. , Adams, P. E. , & Schneiders, B. B. (2017). Stratag: An R package for manipulating, summarizing and analysing population genetic data. Molecular Ecology Resources, 17, 5–11. 10.1111/1755-0998.12559 27327208

[ece38578-bib-0007] Bashalkhanov, S. , Pandey, M. , & Rajora, O. P. (2009). A simple method for estimating genetic diversity in large populations from finite sample sizes. BMC Genetics, 10, 1–10. 10.1186/1471-2156-10-84 20003542 PMC2800116

[ece38578-bib-0008] Bawa, K. S. (1990). Plant‐pollinator interactions in tropical rain forests. Annual Review of Ecology and Systematics, 21, 399–422. 10.1146/annurev.es.21.110190.002151

[ece38578-bib-0009] Bazin, E. , Glémin, S. , & Galtier, N. (2006). Population size does not influence mitochondrial genetic diversity in animals. Science, 312(5773), 570–572. 10.1126/science.1122033 16645093

[ece38578-bib-0010] Bensch, S. , Andrén, H. , Hansson, B. , Pedersen, H. C. , Sand, H. , Sejberg, D. , Wabakken, P. , Åkesson, M. , & Liberg, O. (2006). Selection for heterozygosity gives hope to a wild population of inbred wolves. PLoS One, 1(1), e72. 10.1371/journal.pone.0000072 17183704 PMC1762340

[ece38578-bib-0011] Bowles, M. (1983). The tallgrass prairie orchids *Platanthera leucophaea* (Nutt.) Lindl. and Cypripedium Candidum Muhl. Ex Willd.: Some aspects of their status, biology, and ecology, and implications toward management. Natural Areas Journal. http://plantconservation.us/Bowles1983.pdf

[ece38578-bib-0012] Breed, M. F. , Marklund, M. H. K. , Ottewell, K. M. , Gardner, M. G. , Berton Harris, J. C. , & Lowe, A. J. (2012). Pollen diversity matters: Revealing the neglected effect of pollen diversity on fitness in fragmented landscapes. Molecular Ecology, 21(24), 5955–5968. 10.1111/mec.12056 23078354

[ece38578-bib-0013] Breed, M. F. , Ottewell, K. M. , Gardner, M. G. , Marklund, M. H. K. , Dormontt, E. E. , & Lowe, A. J. (2013). Mating patterns and pollinator mobility are critical traits in forest fragmentation genetics. Heredity, 115(April), 1–7. 10.1038/hdy.2013.48 PMC4815446 24002239

[ece38578-bib-0014] Breed, M. F. , Ottewell, K. M. , Gardner, M. G. , Marklund, M. H. K. , Stead, M. G. , Harris, J. B. C. , & Lowe, A. J. (2015). Mating system and early viability resistance to habitat fragmentation in a bird‐pollinated eucalypt. Heredity, 115(2), 100–107. 10.1038/hdy.2012.72 23188172 PMC4815440

[ece38578-bib-0015] Breed, M. F. , Stead, M. G. , Ottewell, K. M. , Gardner, M. G. , & Lowe, A. J. (2012). Which provenance and where? Seed sourcing strategies for revegetation in a changing environment. Conservation Genetics, 14(1), 1–10. 10.1007/s10592-012-0425-z

[ece38578-bib-0016] Broadhurst, L. M. , & Young, A. G. (2006). Reproductive constraints for the long‐term persistence of fragmented acacia dealbata (Mimosaceae) Populations in Southeast Australia. Biological Conservation, 133(4), 512–526. 10.1016/j.biocon.2006.08.004

[ece38578-bib-0017] Butaye, J. , Jacquemyn, H. , & Hermy, M. (2001). Differential colonization causing non‐random forest plant community structure in a fragmented agricultural landscape. Ecography, 24(4), 369–380. 10.1111/j.1600-0587.2001.tb00472.x

[ece38578-bib-0018] Cabin, R. J. , Mitchell, R. J. , & Marshall, D. L. (1998). Do surface plant and soil seed bank populations differ genetically? A multipopulation study of the desert mustard *Lesquerella fendleri* (Brassicaceae). American Journal of Botany, 85(8), 1098–1109. 10.2307/2446343 21684995

[ece38578-bib-0019] Charlesworth, D. , & Charlesworth, B. (1987). Inbreeding depression and its evolutionary consequences. Annual Review of Ecology and Systematics, 18, 237–268. 10.1146/annurev.es.18.110187.001321

[ece38578-bib-0020] COSEWIC (2003). COSEWIC Assessment and Update Status Report on the Eastern Prairie Fringed‐Orchid *Platanthera leucophaea* in Canada. COSEWIC.

[ece38578-bib-0021] Cuthrell, D. L. , Higman, P. J. , Penskar, M. R. , & Windus, J. L. (1999). The pollinators of Ohio and Michigan populations of eastern prairie fringed orchid (*Platanthera leucophaea*), 1–22.

[ece38578-bib-0022] Del Castillo, R. F. (1994). Factors influencing the genetic structure of *Phacelia dubia*, a species with a seed bank and large fluctuations in population size. Heredity, 72(5), 446–458. 10.1038/hdy.1994.63

[ece38578-bib-0023] Do, C. , Waples, R. S. , Peel, D. , Macbeth, G. M. , Tillett, B. J. , & Ovenden, J. R. (2014). NeEstimator v2: Re‐Implementation of software for the estimation of contemporary effective population size (Ne) from genetic data. Molecular Ecology Resources, 14(1), 209–214. 10.1111/1755-0998.12157 23992227

[ece38578-bib-0024] Doyle, J. J. , & Doyle, J. L. (1987). A rapid DNA isolation procedure for small quantities of fresh leaf tissue. Phytochemical Bulletin, 19, 11–15.

[ece38578-bib-0025] Duminil, J. , Fineschi, S. , & Hampe, A. (2007). Can population genetic structure be predicted from life‐history traits? The American Naturalist, 169(5), 662–672. 10.1086/513490 17427136

[ece38578-bib-0026] Earl, D. A. , & vonHoldt, B. M. (2012). STRUCTURE HARVESTER: A website and program for visualizing STRUCTURE output and implementing the evanno method. Conservation Genetics Resources, 4(2), 359–361. 10.1007/s12686-011-9548-7

[ece38578-bib-0027] England, P. R. , Luikart, G. , & Waples, R. S. (2010). Early detection of population fragmentation using linkage disequilibrium estimation of effective population size. Conservation Genetics, 11(6), 2425–2430. 10.1007/s10592-010-0112-x

[ece38578-bib-0028] Evanno, G. , Regnaut, S. , & Goudet, J. (2005). Detecting the number of clusters of individuals using the software STRUCTURE: A simulation study. Molecular Ecology, 14(8), 2611–2620. 10.1111/j.1365-294X.2005.02553.x 15969739

[ece38578-bib-0029] Fahrig, L. (2017). Ecological responses to habitat fragmentation per se. Annual Review of Ecology, Evolution, and Systematics, 48(November), 1–23. 10.1146/annurev-ecolsys-110316-022612

[ece38578-bib-0030] Fahrig, L. , Arroyo‐Rodríguez, V. , Bennett, J. R. , Boucher‐Lalonde, V. , Cazetta, E. , Currie, D. J. , Eigenbrod, F. , Ford, A. T. , Harrison, S. P. , Jaeger, J. A. G. , Koper, N. , Martin, A. E. , Martin, J.‐L. , Metzger, J. P. , Morrison, P. , Rhodes, J. R. , Saunders, D. A. , Simberloff, D. , Smith, A. C. , … Watling, J. I. (2019). Is habitat fragmentation bad for biodiversity? Biological Conservation, 230, 179–186. 10.1016/j.biocon.2018.12.026

[ece38578-bib-0031] Falush, D. , Stephens, M. , & Pritchard, J. K. (2007). Inference of population structure using multilocus genotype data: Dominant markers and null alleles. Molecular Ecology Notes, 7(4), 574–578. 10.1111/j.1471-8286.2007.01758.x 18784791 PMC1974779

[ece38578-bib-0032] Fisher, R. A. , Steven Corbet, A. , & Williams, C. B. (1943). The relation between the number of species and the number of individuals in a random sample of an animal population. The Journal of Animal Ecology, 12(1), 58. 10.2307/1411

[ece38578-bib-0033] Fletcher, R. J. , Didham, R. K. , Banks‐Leite, C. , Barlow, J. , Ewers, R. M. , Rosindell, J. , Holt, R. D. , Gonzalez, A. , Pardini, R. , Damschen, E. I. , Melo, F. P. L. , Ries, L. , Prevedello, J. A. , Tscharntke, T. , Laurance, W. F. , Lovejoy, T. , & Haddad, N. M. (2018). Is Habitat fragmentation good for biodiversity? Biological Conservation, 226(April), 9–15. 10.1016/j.biocon.2018.07.022

[ece38578-bib-0034] Frankham, R. (2005). Genetics and extinction. Biological Conservation, 126(2), 131–140. 10.1016/j.biocon.2005.05.002

[ece38578-bib-0035] Frankham, R. , Ballou, J. D. , Eldridge, M. D. B. B. , Lacy, R. C. , Ralls, K. , Dudash, M. R. , & Fenster, C. B. (2011). Predicting the probability of outbreeding depression. Conservation Biology, 25(3), 465–475. 10.1111/j.1523-1739.2011.01662.x 21486369

[ece38578-bib-0036] Gilpin, M. E. , & Soule, M. E. (1989). Mininmun viable populations: processes of species extinction. Conservation Biology, 18–19.

[ece38578-bib-0037] González‐Varo, J. P. , Aparicio, A. , Lavergne, S. , Arroyo, J. , & Albaladejo, R. G. (2012). Contrasting heterozygosity‐fitness correlations between populations of a self‐compatible shrub in a fragmented landscape. Genetica, 140(1–3), 31–38. 10.1007/s10709-012-9655-8 22552537

[ece38578-bib-0038] González‐Varo, J. P. , Nora, S. , & Aparicio, A. (2012). Bottlenecks for plant recruitment in woodland remnants: An ornithochorous shrub in a mediterranean ‘relictual’ landscape. Perspectives in Plant Ecology, Evolution and Systematics, 14(2), 111–122. 10.1016/j.ppees.2011.11.002

[ece38578-bib-0039] Gregg, K. B. (1990). The Natural Life Cycle of Platanthera. North American Native Terrestrial Orchid Propagation and Production. Brandywine Conservancy.

[ece38578-bib-0040] Haber, W. A. , & Frankie, G. W. (1989). A tropical Hawkmoth community: Costa Rican dry forest Sphingidae. Biotropica, 21(2), 155–172.

[ece38578-bib-0041] Haddad, N. M. , Brudvig, L. A. , Clobert, J. , Davies, K. F. , Gonzalez, A. , Holt, R. D. , Lovejoy, T. E. et al (2015). Habitat fragmentation and its lasting impact on earth’s ecosystems. Science Advances, 1(2), 1–10. 10.1126/sciadv.1500052 PMC4643828 26601154

[ece38578-bib-0042] Hadley, A. S. , & Betts, M. G. (2016). Refocusing habitat fragmentation research using lessons from the last decade. Current Landscape Ecology Reports, 1(2), 55–66. 10.1007/s40823-016-0007-8

[ece38578-bib-0043] Hale, M. L. , Burg, T. M. , & Steeves, T. E. (2012). Sampling for microsatellite‐based population genetic studies: 25 to 30 individuals per population is enough to accurately estimate allele frequencies. PLoS One, 7(9), e45170. 10.1371/journal.pone.0045170 22984627 PMC3440332

[ece38578-bib-0044] Halkett, F. , Simon, J. C. , & Balloux, F. (2005). Tackling the population genetics of clonal and partially clonal organisms. Trends in Ecology and Evolution, 20(4), 194–201. 10.1016/j.tree.2005.01.001 16701368

[ece38578-bib-0045] Hampe, A. , & Petit, R. J. (2005). Conserving biodiversity under climate change: the rear edge matters. Ecology Letters, 8(5), 461–467. 10.1111/j.1461-0248.2005.00739.x 21352449

[ece38578-bib-0046] Harder, L. (2000). Pollen Dispersal and the Floral Diversity of Monocotyledons. In I. N. Monocots , D. A. Morrison , K. L. Wilson II (Eds.), pp. 243–257. Csiro publishing. Available from https://www.google.com/books/edition/Monocots_Systematics_and_Evolution/YzQBUQqLS0YC?hl=en&gbpv=1&dq=Harder+2000+orchids+pollinaria&pg=PA243&printsec=frontcover

[ece38578-bib-0047] Hardey, O. , & Vekemans, X. (2002). Spagedi: A versatile computer program to analyse spatial genetic structure at the individual or population levels. Molecular Ecology Notes, 2, 618–620. 10.1046/j.1471-8278

[ece38578-bib-0048] Havens, K. , & Alex Buerkle, C. (1999). A Population Genetic Analysis of *Platanthera leucophaea* in Northeastern Illinois. *Report to US Fish and Wildlife Service*.

[ece38578-bib-0049] Hoebee, S. E. , Thrall, P. H. , & Young, A. G. (2007). Integrating population demography, genetics and self‐incompatibility in a viability assessment of the Wee Jasper Grevillea (Grevillea Iaspicula McGill., Proteaceae). Conservation Genetics, 9(3), 515–529. 10.1007/s10592-007-9366-3

[ece38578-bib-0050] Honnay, O. , Bossuyt, B. , Jacquemyn, H. , Shimono, A. , & Uchiyama, K. (2008). Can a seed bank maintain the genetic variation in the above ground plant population? Oikos, 117(1), 1–5. 10.1111/j.2007.0030-1299.16188.x

[ece38578-bib-0051] Honnay, O. , & Jacquemyn, H. (2007a). Susceptibility of Common and Rare Plant Species to the Genetic Consequences of Habitat Fragmentation. Conservation Biology, 21(3), 823–831. 10.1111/j.1523-1739.2006.00646.x 17531059

[ece38578-bib-0052] Honnay, O. , & Jacquemyn, H. (2007b). Susceptibility of common and rare plant species to the genetic consequences of habitat fragmentation. Conservation Biology, 21(3), 823–831. 10.1111/j.1523-1739.2006.00646.x 17531059

[ece38578-bib-0053] Honnay, O. , Verheyen, K. , Butaye, J. , Jacquemyn, H. , Bossuyt, B. , & Hermy, M. (2002). Possible effects of habitat fragmentation and climate change on the range of forest plant species. Ecology Letters, 5(4), 525–530. 10.1046/j.1461-0248.2002.00346.x

[ece38578-bib-0054] Ibáñez, I. , Katz, D. S. W. , Peltier, D. , Wolf, S. M. , & Connor Barrie, B. T. (2014). Assessing the integrated effects of landscape fragmentation on plants and plant communities: the challenge of Multiprocess‐Multiresponse dynamics. Journal of Ecology, 102(4), 882–895. 10.1111/1365-2745.12223

[ece38578-bib-0055] Jacquemyn, H. , Honnay, O. , Galbusera, P. , & Roldan‐Ruiz, I. (2004). Genetic structure of the forest Herb *Primula elatior* in a changing landscape. Molecular Ecology, 13(1), 211–219. 10.1046/j.1365-294X.2003.02033.x 14653801

[ece38578-bib-0056] Johnson, M. G. , & Jonathan Shaw, A. (2015). Genetic diversity, sexual condition, and microhabitat preference determine mating *Patterns in sphagnum* (Sphagnaceae) Peat‐mosses. Biological Journal of the Linnean Society, 115(1), 96–113. 10.1111/bij.12497

[ece38578-bib-0057] Johnson, S. D. , Neal, P. R. , & Harder, L. D. (2005). Pollen fates and the limits on male reproductive success in an orchid population. Biological Journal of the Linnean Society, 86(2), 175–190. 10.1111/j.1095-8312.2005.00541.x

[ece38578-bib-0127] Jombart, T. (2008). Adegenet: A R package for the multivariate analysis of genetic markers. Bioinformatics, 24(11), 1403–1405. 10.1093/bioinformatics/btn129 18397895

[ece38578-bib-0058] Juárez, L. , Montaña, C. , & Ferrer, M. M. (2011). Genetic structure at patch level of the terrestrial orchid *Cyclopogon luteoalbus* (Orchidaceae) in a fragmented cloud forest. Plant Systematics and Evolution, 297(3–4), 237–251. 10.1007/s00606-011-0511-6

[ece38578-bib-0059] Juillet, N. , Dunand‐Martin, S. , & Gigord, L. D. B. (2007). Evidence for inbreeding depression in the food‐deceptive colour‐dimorphic orchid *Dactylorhiza sambucina* (L.) Soò. Plant Biology, 9(1), 147–151. 10.1055/s-2006-924310 16883478

[ece38578-bib-0060] Jump, A. S. , Marchant, R. , & Peñuelas, J. (2009). Environmental change and the option value of genetic diversity. Trends in Plant Science, 14(1), 51–58. 10.1016/j.tplants.2008.10.002 19042147

[ece38578-bib-0125] Kassambara, A. , & Mundt, F. (2020). Factoextra: Extract and visualize the results of multivariate data analyses. R package version 1.0.7. https://CRAN.R‐project.org/package=factoextra

[ece38578-bib-0061] Kramer, A. T. , Ison, J. L. , Ashley, M. V. , & Howe, H. F. (2008). The paradox of forest fragmentation genetics. Conservation Biology, 22(4), 878–885. 10.1111/j.1523-1739.2008.00944.x 18544089

[ece38578-bib-0062] Lande, R. , & Shannon, S. (1996). The role of genetic variation in adaptation and population persistence in a changing environment. Evolution, 50(1), 434–437. 10.1111/j.1558-5646.1996.tb04504.x 28568879

[ece38578-bib-0063] Leimu, R. , Mutikainen, P. , Koricheva, J. , & Fischer, M. (2006). How general are positive relationships between plant population size, fitness and genetic variation? Journal of Ecology, 94(5), 942–952. 10.1111/j.1365-2745.2006.01150.x

[ece38578-bib-0064] Leimu, R. , Vergeer, P. , Angeloni, F. , & Joop Ouborg, N. (2010). Habitat fragmentation, climate change, and inbreeding in plants. Annals of the New York Academy of Sciences, 1195(May), 84–98. 10.1111/j.1749-6632.2010.05450.x 20536818

[ece38578-bib-0065] Lienert, J. (2004). Habitat fragmentation effects of fitness of plant populations ‐ A review. Journal for Nature Conservation, 12(1), 53–72. 10.1016/j.jnc.2003.07.002

[ece38578-bib-0066] Linhart, Y. B. , & Mendenhall, J. A. (1977). Pollen Dispersal by Hawkmoths in a Lindenia Rivalis Benth. Population in Belize. Biotropica, 9, 143. 10.2307/2387672

[ece38578-bib-0067] Loveless, M. D. , & Hamrick, J. L. (1984). Ecological determinants of genetic structure in plant populations. Annual Review of Ecology and Systematics, 15, 65–95. 10.1146/annurev.es.15.110184.000433

[ece38578-bib-0068] Lowe, A. J. , Boshier, D. , Ward, M. , Bacles, C. F. E. , & Navarro, C. (2005). Genetic resource impacts of habitat loss and degradation; reconciling empirical evidence and predicted theory for Neotropical trees. Heredity, 95(4), 255–273. 10.1038/sj.hdy.6800725 16094300

[ece38578-bib-0069] Lowe, W. H. , Kovach, R. P. , & Allendorf, F. W. (2017). Population genetics and demography unite ecology and evolution. Trends in Ecology and Evolution, 32(2), 141–152. 10.1016/j.tree.2016.12.002 28089120

[ece38578-bib-0070] Luyt, R. , & Johnson, S. D. (2001). Hawkmoth pollination of the African epiphytic orchid *Mystacidium venosum*, with special reference to flower and pollen longevity. Plant Systematics and Evolution, 228, 49–62. 10.1007/s006060170036 .

[ece38578-bib-0071] Mace, G. M. , & Purvis, A. (2008). Evolutionary biology and practical conservation: Bridging a widening gap. Molecular Ecology, 17(1), 9–19. 10.1111/j.1365-294X.2007.03455.x 17696991

[ece38578-bib-0072] Machon, N. , Bardin, P. , Mazer, S. J. , Moret, J. , Godelle, B. , & Austerlitz, F. (2003). Relationship between genetic structure and seed and pollen dispersal in the endangered orchid *Spiranthes spiralis* . New Phytologist, 157(3), 677–687. 10.1046/j.1469-8137.2003.00694.x 33873401

[ece38578-bib-0073] Manel, S. , & Holderegger, R. (2013). Ten years of landscape genetics. Trends in Ecology and Evolution, 28(10), 614–621. 10.1016/j.tree.2013.05.012 23769416

[ece38578-bib-0074] Matesanz, S. , Teso, M. L. R. , García‐Fernández, A. , & Escudero, A. (2017). Habitat fragmentation differentially affects genetic variation, phenotypic plasticity and survival in populations of a gypsum endemic. Frontiers in Plant Science, 8(843). 10.3389/fpls.2017.00843 PMC5445106 28603529

[ece38578-bib-0075] Miles, L. S. , Ruth Rivkin, L. , Johnson, M. T. J. , Munshi‐South, J. , & Verrelli, B. C. (2019). Gene flow and genetic drift in urban environments. Molecular Ecology, 28(18), 4138–4151. 10.1111/mec.15221 31482608

[ece38578-bib-0076] Nilsson, L. A. (1983). Processes of isolation and introgressive interplay between *Platanthera bifolia* (L.) Rich and *P. Chlorantha* (Custer) Reichb. (Orchidaceae). Botanical Journal of the Linnean Society, 87(4), 325–350. 10.1111/j.1095-8339.1983.tb00997.x

[ece38578-bib-0077] Nilsson, L. A. (1992). Orchid pollination biology. Trends in Ecology and Evolution, 7(8), 255–259. 10.1016/0169-5347(92)90170-G 21236024

[ece38578-bib-0078] Nilsson, L. A. , Johnsson, L. , Ralison, L. , & Randrianjohany, E. (2009). Angraecoid orchids and hawkmoths in central madagascar : specialized pollination systems and generalist foragers published by : the association for tropical biology and conservation Stable URL : http://www.jstor.org/stable/2388628, 19(4), 310–18.

[ece38578-bib-0079] Oakley, C. G. , Ågren, J. , & Schemske, D. W. (2015). Heterosis and outbreeding depression in crosses between natural populations of Arabidopsis Thaliana. Heredity, 115(1), 73–82. 10.1038/hdy.2015.18 26059971 PMC4815493

[ece38578-bib-0080] Oakley, C. G. , Lundemo, S. , Ågren, J. , & Schemske, D. W. (2019). Heterosis is common and inbreeding depression absent in natural populations of Arabidopsis Thaliana. Journal of Evolutionary Biology, 32(6), 592–603. 10.1111/jeb.13441 30883966

[ece38578-bib-0128] Oksanen, J. , Blanchet, F. G. , Friendly, M. , Kindt, R. , Legendre, P. , McGlinn, D. , Minchin, P. R. , O’Hara, R. B. , Simpson, G. L. , Solymos, P. , Henry, M. , Stevens, H. , Szoecs, E. , & Wagner, H. (2019). vegan: Community ecology package. R package version 2.5‐7. https://CRAN.R‐project.org/package=vegan

[ece38578-bib-0081] Oostermeijer, J. G. B. , Van Eijck, M. W. , Van Leeuwen, N. C. , & Den Nijs, J. C. M. (1995). Analysis of the relationship between *Allozyme heterozygosity* and fitness in the rare *Gentiana pneumonanthe* L. Journal of Evolutionary Biology, 8(6), 739–759. 10.1046/j.1420-9101.1995.8060739.x

[ece38578-bib-0082] Ortiz‐Barney, E. , & Ackerman, J. D. (1999). The cost of selfing in *Encyclia cochleata* (Orchidaceae). Plant Systematics and Evolution, 219(1–2), 55–64. 10.1007/BF01090299

[ece38578-bib-0083] Parmesan, C. (2006). Ecological and evolutionary responses to recent climate change. Annual Review of Ecology, Evolution, and Systematics, 37(1), 637–669. 10.1146/annurev.ecolsys.37.091305.110100

[ece38578-bib-0084] Patt, J. M. , Merchant, M. W. , Williams, D. R. E. , Bastiaan, J. D. , Patt, J. M. , Merchant, M. W. , Williams, D. R. E. , & Meeuse, B. J. D. (1989). Pollination biology of *Platanthera stricta* (Orchidaceae) in Olympic national Park. American Journal of Botany, 76(8), 1097–1106.

[ece38578-bib-0085] Paul, J. , Budd, C. , & Freeland, J. R. (2013). Conservation genetics of an endangered orchid in Eastern Canada. Conservation Genetics, 14(1), 195–204. 10.1007/s10592-012-0443-x

[ece38578-bib-0086] Pavlova, A. S. , Leontieva, M. R. , Smirnova, T. A. , Kolomeitseva, G. L. , Netrusov, A. I. , & Tsavkelova, E. A. (2017). Colonization strategy of the endophytic plant growth‐promoting strains of *Pseudomonas fluorescens* and *Klebsiella oxytoca* on the seeds, seedlings and roots of the epiphytic orchid, *Dendrobium nobile* lindl. Journal of Applied Microbiology, 123(1), 217–232. 10.1111/jam.13481 28457004

[ece38578-bib-0087] Peakall, R. (1989). A New Technique for Monitoring Pollen Flow in Orchids. Oecologia, 79(3), 361–365. 10.1007/BF00384315 23921401

[ece38578-bib-0088] Peakall, R. , & Smouse, P. E. (2006). GENALEX 6: Genetic analysis in excel. population genetic software for teaching and research. Molecular Ecology Notes, 6(1), 288–295. 10.1111/j.1471-8286.2005.01155.x PMC3463245 22820204

[ece38578-bib-0089] Phillips, R. D. , Dixon, K. W. , & Peakall, R. (2012). Low population genetic differentiation in the Orchidaceae: Implications for the diversification of the family. Molecular Ecology, 21(21), 5208–5220. 10.1111/mec.12036 23017205

[ece38578-bib-0090] Pleasants, J. M. , & Klier, K. (1995). Genetic variation within and among populations of the eastern and western prairie fringed orchids, *Platanthera leucophaea* and *P. Praeclara* . Available from https://scholar.google.com/scholar?hl=en&as_sdt=0%2C48&q=JM+Pleasants%2C+K+Klier+‐+Report+to+the+Iowa+DNR%2C+1995&btnG=

[ece38578-bib-0091] Pons, O. , & Petit, R. J. (1996). Measuring and testing genetic differentiation with Ordered vs. Unordered alleles. Genetics 144 (May 2014): 1237–1245. 8913764 10.1093/genetics/144.3.1237 PMC1207615

[ece38578-bib-0092] Pritchard, J. K. , Stephens, M. , & Donnelly, P. (2000). Inference of population structure using multilocus genotype data. Genetics, 155(2), 945–959. 10.1111/j.1471-8286.2007.01758.x 10835412 PMC1461096

[ece38578-bib-0093] R CoreTeam . (2016). R: A Language and Environment for Statistical Computing. R Foundation for Statistical Computing.

[ece38578-bib-0094] Ralls, K. , Ballou, J. D. , Dudash, M. R. , Eldridge, M. D. B. , Fenster, C. B. , Lacy, R. C. , Sunnucks, P. , & Frankham, R. (2018). Call for a paradigm shift in the genetic management of fragmented populations. Conservation Letters, 11(2), 1–6. 10.1111/conl.12412

[ece38578-bib-0095] Rasmussen, D. I. (1979). Sibling clusters and genotypic frequencies. American Society of Naturalists, 113(6), 948–951. 10.1086/283449

[ece38578-bib-0096] Raymond, G. , & Rousset, F. (1995). GENEPOP (Version 1.2): Population genetics software for exact tests and Ecumenicism. Heredity, 86(3), 248–249. 10.1093/oxfordjournals.jhered.a111573

[ece38578-bib-0097] Reed, D. H. , & Frankham, R. (2003). Correlation between fitness and genetic diversity. Conservation Biology, 17(1), 230–237. 10.1046/j.1523-1739.2003.01236.x

[ece38578-bib-0098] Richards, C. M. (2000). Inbreeding depression and genetic rescue in a Plant Metapopulation. American Naturalist, 155(3), 383–394. 10.1086/303324 10718733

[ece38578-bib-0099] Ross, A. A. , Aldrich‐Wolfe, L. , Lance, S. , Glenn, T. , & Travers, S. E. (2013). Microsatellite markers in the western prairie fringed orchid, *Platanthera praeclara* (Orchidaceae). Applications in Plant Sciences, 1(4), 1–4. 10.3732/apps.1200413 PMC4105293 25202536

[ece38578-bib-0100] Ross, A. A. , & Travers, S. E. (2016). The genetic consequences of rarity in the western prairie fringed orchid (*Platanthera praeclara*). Conservation Genetics, 17(1), 69–76. 10.1007/s10592-015-0761-x

[ece38578-bib-0101] Rousset, F. (1997). Genetic differentiation and estimation of gene flow from statistics under isolation by distance franqois. Genetics Society of America, 145, 1219–1228. 10.1002/ajmg.c.30221 PMC1207888 9093870

[ece38578-bib-0102] Salguero‐faria, J. A. , & Ackerman, J. D. (1999). A nectar reward: is more better? Biotropica, 31(2), 303–311.

[ece38578-bib-0103] Schlaepfer, D. R. , Braschler, B. , Rusterholz, H.‐P. , & Baur, B. (2018). Genetic effects of anthropogenic habitat fragmentation on remnant animal and plant populations: A meta‐analysis. Ecosphere, 9(10), e02488. 10.1002/ecs2.2488

[ece38578-bib-0104] Shah, V. B. , & McRae, B. H. (2008). Circuitscape: A tool for landscape ecology. In Proceedings of the 7th Python in Science Conference, pp. 62–65. 10.1111/j.1523-1739.2008.00942.x

[ece38578-bib-0105] Shefferson, R. P. , Bunch, W. , Cowden, C. C. , Lee, Y. I. , Kartzinel, T. R. , Yukawa, T. , Downing, J. , & Jiang, H. (2019). Does evolutionary history determine specificity in broad ecological interactions? Journal of Ecology, 107(4), 1582–1593. 10.1111/1365-2745.13170

[ece38578-bib-0106] Skogen, K. A. , Overson, R. P. , Hilpman, E. T. , & Fant, J. B. (2019). Hawkmoth pollination facilitates long‐distance pollen dispersal and reduces isolation across a gradient of land‐use change. Annals of the Missouri Botanical Garden, 104(3), 495–511. 10.3417/2019475

[ece38578-bib-0107] Sletvold, N. , Grindeland, J. M. , Pengjuan, Z. U. , & Ågren, J. (2012). Strong inbreeding depression and local outbreeding depression in the rewarding orchid *Gymnadenia conopsea* . Conservation Genetics, 13(5), 1305–1315. 10.1007/s10592-012-0373-7

[ece38578-bib-0108] Spielman, D. , Brook, B. W. , & Frankham, R. (2004). Most species are not drive to extinction before genetic factors impact them. PNAS, 101(42), 15261–15264. 15477597 10.1073/pnas.0403809101 PMC524053

[ece38578-bib-0109] Stilwell, K. L. , Wilbur, H. M. , Werth, C. R. , & Taylor, D. R. (2003). Heterozygote advantage in the American chestnut, *Castanea dentata* (Fagaceae). American Journal of Botany, 90(2), 207–213. 10.3732/ajb.90.2.207 21659110

[ece38578-bib-0110] Stoeckel, S. , Grange, J. , Fernández‐Manjarres, J. F. , Bilger, I. , Frascaria‐Lacoste, N. , & Mariette, S. (2006). Heterozygote excess in a self‐incompatible and partially clonal forest tree species ‐ *Prunus avium* L. Molecular Ecology, 15(8), 2109–2118. 10.1111/j.1365-294X.2006.02926.x 16780428

[ece38578-bib-0111] Sujii, P. S. , Martins, K. , Wadt, L. H. D. O. , Azevedo, V. C. R. , & Solferini, V. N. (2015). Genetic structure of *Bertholletia excelsa* populations from the amazon at different spatial scales. Conservation Genetics, 16(4), 955–964. 10.1007/s10592-015-0714-4

[ece38578-bib-0112] Tonsor, S. , Kalisz, S. , Fisher, J. , & Holtsford, T. (1993). Life history based study of population genetic structure: seed bank to adult sin *Plantago lanceolata* . Evolution, 47(3), 833–843. 28567912 10.1111/j.1558-5646.1993.tb01237.x

[ece38578-bib-0113] Travers, S. E. , Anderson, K. , Vitt, P. , & Harris, M. O. (2018). Breeding system and inbreeding depression in the rare orchid, *Platanthera praeclara*, in a fragmented grassland landscape. Botany‐Botanique, 96(3), 151–159. 10.1139/cjb-2017-0104

[ece38578-bib-0114] Tremblay, R. L. (1994). Frequency and consequences of multi‐parental pollinations in a populations of cypripedium Calceolus Var. Pubescens (Orchidaceae). Lindleyana, 9(3), 161–167.

[ece38578-bib-0115] USFWS . (1999). Eastern Prairie Fringed Orchid, *Platanthera leucophaea* (Nuttall) Lindley Recovery Plan.

[ece38578-bib-0116] USFWS . (2016). Eastern Prairie Fringed Orchid (*Platanthera leucophaea*) 5‐Year Review: Summary and Evaluation. USFWS Chicago Field Office.

[ece38578-bib-0117] Van Oosterhout, C. , Hutchinson, W. F. , Wills, D. P. M. , & Shipley, P. (2004). MICRO‐CHECKER: Software for identifying and correcting genotyping errors in microsatellite data. Molecular Ecology Notes, 4(3), 535–538. 10.1111/j.1471-8286.2004.00684.x

[ece38578-bib-0118] Vilas, C. , Miguel, E. S. , Amaro, R. , & Garcia, C. (2006). Relative contribution of inbreeding depression and eroded adaptive diversity to extinction risk in small populations of shore campion\rContribución Relativa de La Depresión Endogámica y La Diversidad Adaptativa Erosionada Al Riesgo de Extinción de Poblaci. Conservation Biology, 20(1), 229–238. 10.1111/j.1523-1739.2005.00275.x 16909676

[ece38578-bib-0119] Vranckx, G. , Jacquemyn, H. , Muys, B. , & Honnay, O. (2011). Meta‐Analysis of Susceptibility of Woody Plants to Loss of Genetic Diversity through Habitat Fragmentation. Conservation Biology, 26(2), 228–237. 10.1111/j.1523-1739.2011.01778.x 22044646

[ece38578-bib-0120] Wallace, L. E. (2002). Examining the effects of fragmentation on genetic variation in *Platanthera leucophaea* (Orchidaceae): Inferences from allozyme and random amplified polymorphic DNA markers. Plant Species Biology, 17(1), 37–49. 10.1046/j.1442-1984.2002.00072.x

[ece38578-bib-0121] Wallace, L. E. (2003). The cost of Inbreeding in *Platanthera leucophaea* (Orchidaceae). American Journal of Botany, 90(2), 235–242. 10.3732/ajb.90.2.235 21659113

[ece38578-bib-0126] Waples, R. S. , & Do, C. (2008). LDNE: A program for estimating effective population size from data on linkage disequilibrium. Molecular Ecology Resources, 8(4), 753–756. 10.1111/j.1755-0998.2007.02061.x 21585883

[ece38578-bib-0122] Whiteley, A. R. , Fitzpatrick, S. W. , Chris Funk, W. , & Tallmon, D. A. (2014). Genetic rescue to the Rescue. Trends in Ecology and Evolution, 30(1), 42–49. 10.1016/j.tree.2014.10.009 25435267

[ece38578-bib-0123] Yates, C. J. , Elliott, C. , Byrne, M. , Coates, D. J. , & Fairman, R. (2007). Seed production, germinability and seedling growth for a bird‐pollinated Shrub in fragments of Kwongan in South‐West Australia. Biological Conservation, 136(2), 306–314. 10.1016/j.biocon.2006.12.003

[ece38578-bib-0124] Zettler, L. W. , & Piskin, K. A. (2011). *Mycorrhizal fungi* from Protocorms, Seedlings and mature plants of the eastern prairie fringed Orchid, *Platanthera leucophaea* (Nutt.) Lindley: A comprehensive list to augment conservation. American Midland Naturalist, 166(1), 29–39. 10.1674/0003-0031-166.1.29

