# Supplementary material for: Anthropogenic fragmentation increases risk of genetic decline in the threatened orchid Platanthera leucophaea
Source: Ecol Evol. 2022 Feb 17;12(2):e8578. doi: 10.1002/ece3.8578 (PMC8855017; doi:10.1002/ece3.8578)
Supplement: Supplementary file 1 — Supplementary Material [file ECE3-12-e8578-s001.docx]

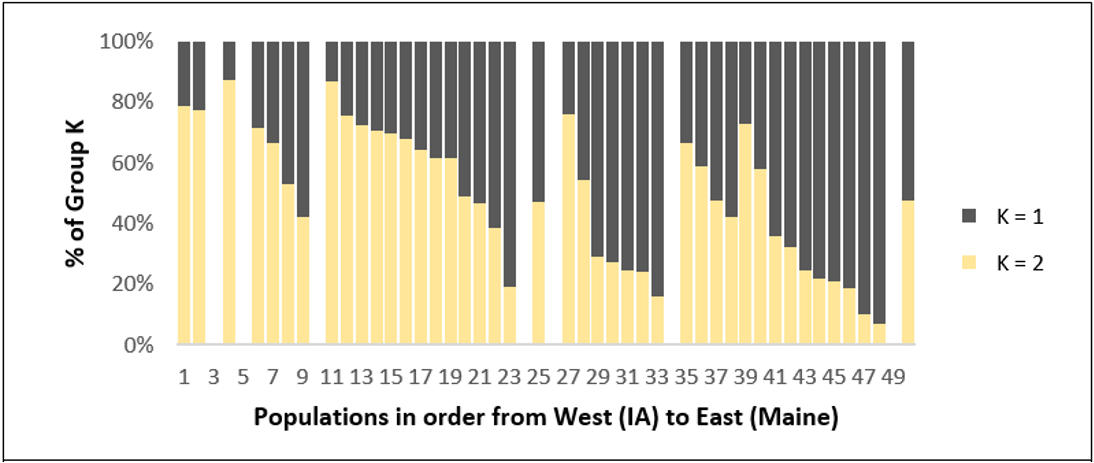


**Supplemental Figure 1**. Identified genetic cluster (1&2) and Bayesian admixture proportions depicted for individual plants and populations spanning the complete range of *Platanthera leucophaea*.


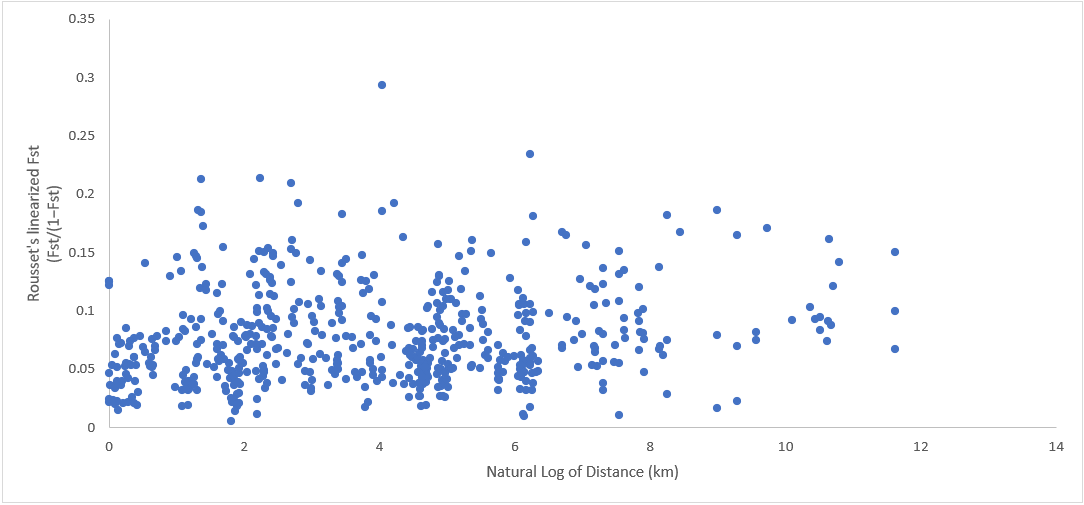


**Supplemental Figure 2.** Pairwise F_st_ by Euclidean geographic distance for all population pairs (samples size >20).


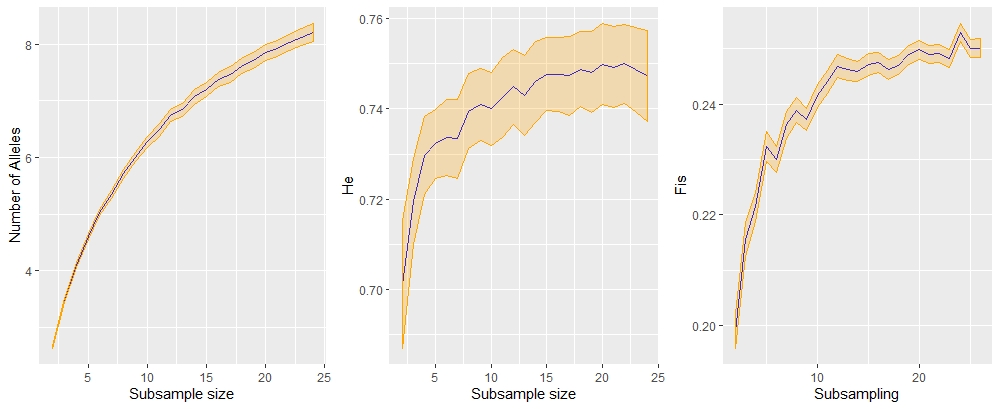


A

A

CA

B

A

**Supplemental Figure 3.** Mean standard error of number of alleles(Na) (a), heterozygosity(He) (b), inbreeding(F_is_) (c) compared to subsample size.

**
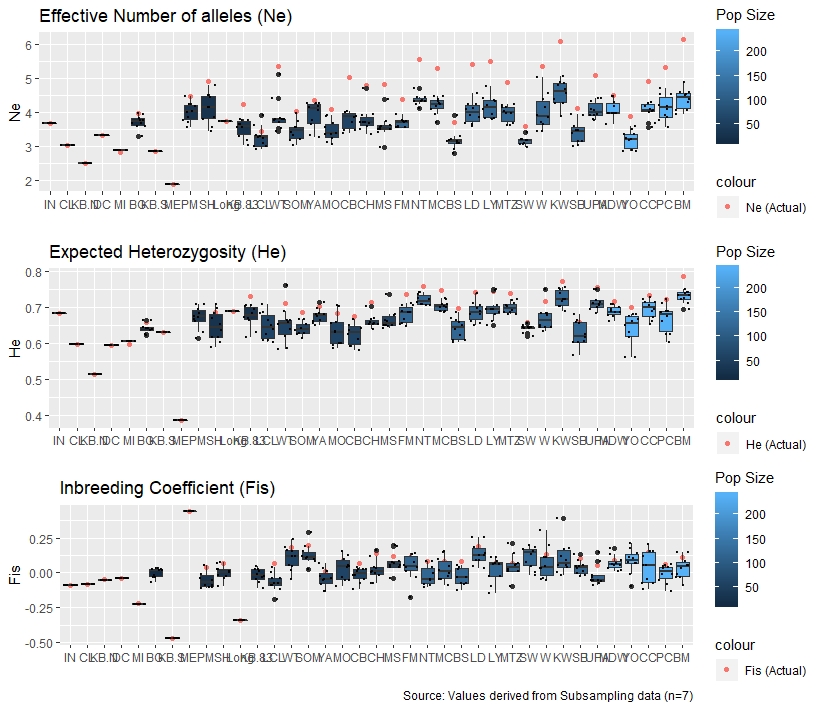
Supplemental Figure 4.** Genetic parameters Effective number of alleles (Ne), Expected heterozygosity (He), and Inbreeding coefficient (F_is_), by population, randomly subsampled to represent critically small populations (n=7). Box plots represent the range of genetic parameter values for subsamples, error bars represent standard deviation, and red dots represent the actual value of the genetic parameters of the complete sample size.

B

A

C

A

A

A

**Supplemental table 1.** Population size data for all populations of *Platanthera leucophaea*, categorical population size, minimum, maximum, median population size at time of census, and effective population size (Neff).

| Code | State | Census 2015 | Cat Pop Size | Min CensusSize | Max Census Size | Med Census Size | NeffPos | NeffMin | NeffMax |
| --- | --- | --- | --- | --- | --- | --- | --- | --- | --- |
| BG* | MI | 10 | 1 | 10 | 10 | 10 | 1000 | 20 | 1000 |
| BM | IA | 600 | 4 | 600 | 600 | 600 | 56.8 | 30 | 220 |
| BS | IL | 30 | 4 | 48 | 133 | 64 | 112.9 | 29 | 1000 |
| CB | WI | 75 | 4 | 75 | 75 | 75 | 1000 | 1 | 1000 |
| CC | OH | 654 | 4 | 126 | 654 | 216 | 81.6 | 36 | 1000 |
| CH | WI | 75 | 1 | 75 | 75 | 75 | 95.6 | 38 | 1000 |
| DC | MI | 7 | 1 | 7 | 7 | 7 | 31.2 | 7 | 1000 |
| FM | WI | 63 | 4 | 63 | 63 | 63 | 44.8 | 23 | 183 |
| GC | IL | 110 | 3 | 2 | 110 | 17 | 12.5 | 8 | 20 |
| IN | IN | 5 | 1 | 5 | 5 | 5 | 9.7 | 4 | 1000 |
| KB.83 | OH | 68 | 2 | 1 | 94 | 10.5 | 115.4 | 38 | 1000 |
| KB.N | OH | 8 | 1 | 1 | 13 | 7 | 63.8 | 2 | 1000 |
| KB.S | OH | 6 | 2 | 1 | 22 | 9.5 | 1.8 | 1 | 1000 |
| KW | WI | 100 | 4 | 100 | 100 | 100 | 62.6 | 31 | 397 |
| LCL | MI | 38 | 2 | 38 | 38 | 38 | 40.5 | 22 | 120 |
| LD | OH | 132 | 4 | 10 | 132 | 81 | 1000 | 64 | 1000 |
| LG | IL | 43 | 3 | 2 | 94 | 30 | 21.2 | 14 | 37 |
| Long | IL | 6 | 1 | 3 | 104 | 18 | 12.7 | 12 | 1000 |
| LY | IL | 103 | 4 | 2 | 200 | 55 | 57.6 | 31 | 214 |
| MC | IL | 39 | 4 | 1 | 744 | 84.5 | 341.4 | 58 | 1000 |
| MDW* | OH | 15 | 4 | 8 | 633 | 101 | 22.5 | 10 | 289 |
| ME | ME | 15 | 2 | 15 | 15 | 15 | 3.7 | 1 | 1000 |
| MI | MI | 7 | 1 | 7 | 7 | 7 | 11.7 | 41 | 1000 |
| MO | MO | 57 | 3 | 57 | 57 | 57 | 107.1 | 35 | 1000 |
| MS | IA | 75 | 2 | 75 | 75 | 75 | 38.4 | 21 | 106 |
| MTZ* | OH | 25 | 2 | 1 | 496 | 17 | 82.2 | 29 | 1000 |
| NG | IL | 425 | 4 | 3 | 425 | 45 | 59.3 | 26 | 1000 |
| NT | OH | 135 | 4 | 2 | 248 | 59 | 46.5 | 24 | 205 |
| PC | OH | 334 | 4 | 1 | 5605 | 111 | 1000 | 64 | 1000 |
| PM | MI | 18 | 1 | 18 | 18 | 18 | 434.2 | 33 | 1000 |
| SB | MI | 100 | 4 | 100 | 100 | 100 | 112.8 | 41 | 1000 |
| SOM | IL | 394 | 4 | 1 | 394 | 10 | 27.1 | 16 | 58 |
| SP | IL | 9 | 1 | 2 | 10 | 7 | 81.3 | 9 | 1000 |
| SW | MI | 158 | 4 | 158 | 158 | 158 | 25.6 | 9 | 1000 |
| UPA | WI | 100 | 4 | 100 | 100 | 100 | 33.8 | 21 | 71 |
| W | IL | 347 | 4 | 1 | 347 | 60 | 43.7 | 24 | 129 |
| WT | IL | 33 | 3 | 1 | 154 | 21.5 | 38.4 | 21 | 116 |
| YA* | OH | 23 | 1 | 1 | 193 | 35 | 1000 | 56 | 1000 |
| YO | OH | 1316 | 3 | 12 | 1316 | 104 | 36.9 | 19 | 127 |
| * Census data for these populations extends beyond date of leaf sampling (1998/1999) | | | | | | | | | |

**Supplemental Table 2.** Landcover composition from the 2011 Gap analysis national landcover data, of 1km buffers around each population of *Platanthera leucophaea* used to calculate patch area.


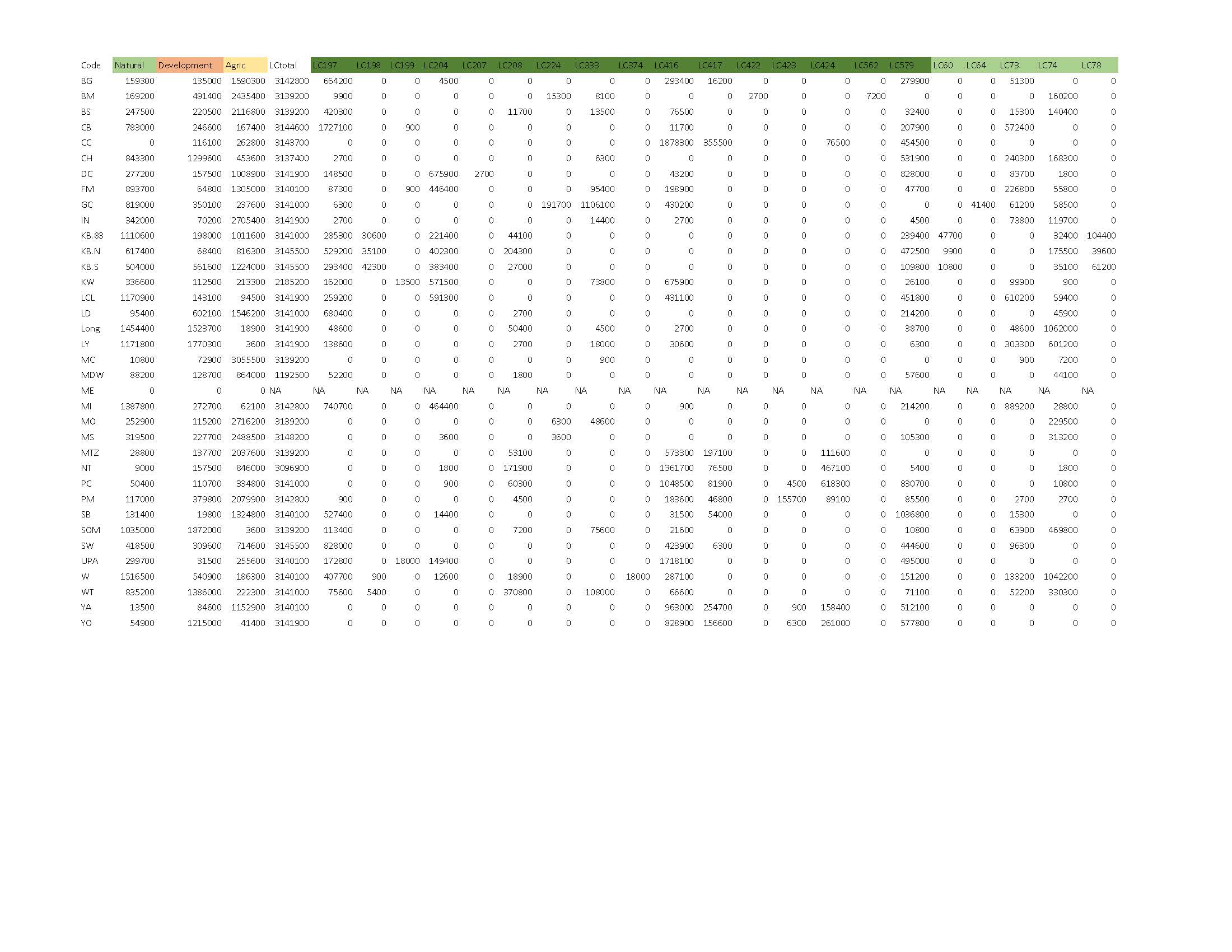


**Supplemental Table 2. Cont.**


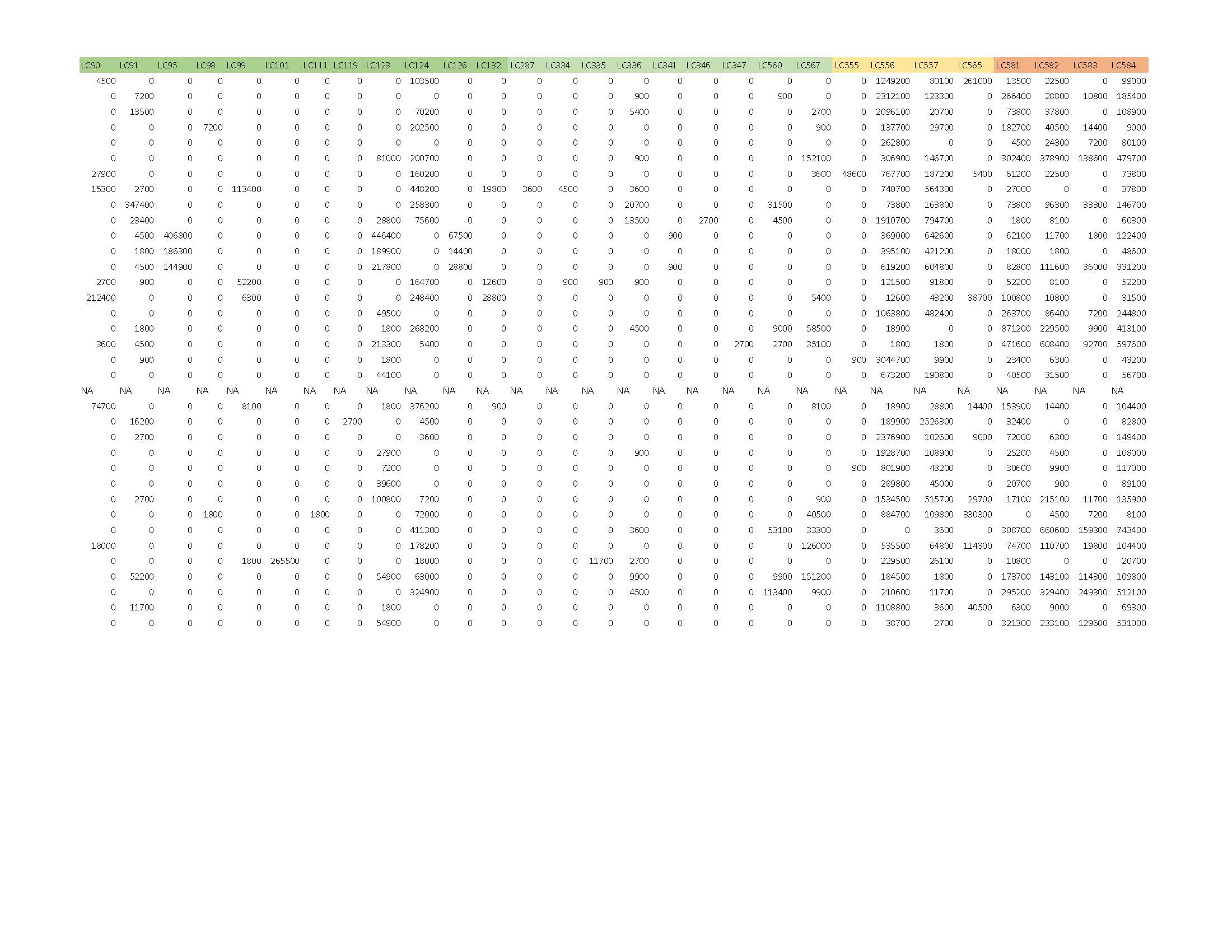


**Supplemental Table 3.** Isolation results calculated in Circuitscape (Resistance), and nearest 5th and 10th neighbor (NeN5, NeN10) calculated in ArcMap, and the average Euclidian pairwise distance calculated in SPAGeDi for each population of *Platanthera leucophaea.*

| Code | State | NeN5 | NeN10 | Resistance | Distance |
| --- | --- | --- | --- | --- | --- |
| BG | MI | 0.21 | 0.68 | 3.22 | 4.19 |
| BM | IA | 1.03 | 1.50 | 3.40 | 5.55 |
| BS | IL | 0.28 | 0.39 | 3.18 | 3.83 |
| CB | WI | 0.83 | 0.90 | 2.78 | 3.96 |
| CC | OH | 0.13 | 0.24 | 2.88 | 3.89 |
| CH | WI | 0.12 | 0.16 | 2.98 | 3.59 |
| DC | MI | 1.23 | 1.51 | 3.10 | 3.37 |
| FM | WI | 0.29 | 0.46 | 2.65 | 4.21 |
| GC | IL | 0.48 | 0.54 | 3.10 | 3.84 |
| IN | IN | 1.22 | 1.36 | 3.18 | 3.68 |
| KB.83 | OH | 0.81 | 1.21 | 3.32 | 4.99 |
| KB.N | OH | 0.78 | 1.16 | 3.06 | 4.99 |
| KB.S | OH | 0.82 | 1.23 | 3.19 | 4.99 |
| KW | WI | 0.29 | 0.47 | 2.85 | 4.22 |
| LCL | MI | 0.79 | 0.93 | 2.66 | 3.61 |
| LD | OH | 1.55 | 1.73 | 3.43 | 4.31 |
| LG | IL | 0.41 | 0.50 | 2.99 | 3.90 |
| Long | IL | 0.14 | 0.16 | 2.82 | 3.61 |
| LY | IL | 0.06 | 0.09 | 2.84 | 3.57 |
| MC | IL | 1.22 | 1.47 | 3.59 | 5.09 |
| MDW* | OH | 1.76 | 1.97 | 3.35 | 4.31 |
| ME | ME | 8.46 | 11.16 | NA | 18.38 |
| MI | MI | 1.29 | 1.65 | 2.60 | 3.45 |
| MO | MO | 2.67 | 3.82 | 4.08 | 8.28 |
| MS | IA | 1.15 | 1.68 | 3.52 | 5.81 |
| MTZ | OH | 0.16 | 0.34 | 2.92 | 4.04 |
| NG | IL | 0.79 | 0.93 | 2.98 | 4.40 |
| NT | OH | 0.15 | 0.33 | 2.83 | 4.03 |
| PC | OH | 0.18 | 0.36 | 2.87 | 4.07 |
| PM | MI | 0.24 | 0.33 | 3.29 | 3.91 |
| SB | MI | 0.29 | 0.76 | 3.22 | 4.32 |
| SOM | IL | 0.07 | 0.12 | 2.92 | 3.56 |
| SP | IL | 0.18 | 0.23 | 3.34 | 3.64 |
| SW | MI | 0.36 | 0.82 | 3.20 | 4.39 |
| UPA | WI | 0.69 | 0.95 | 2.98 | 4.63 |
| W | IL | 0.08 | 0.10 | 2.72 | 3.61 |
| WT | IL | 0.05 | 0.08 | 2.85 | 3.58 |
| YA | OH | 0.13 | 0.26 | 2.97 | 3.86 |
| YO | OH | 0.16 | 0.30 | 3.01 | 4.02 |
